# *Babesia divergens* host cell egress is mediated by essential and druggable kinases and proteases

**DOI:** 10.1038/s41564-025-02238-7

**Published:** 2026-01-27

**Authors:** Brendan Elsworth, Caroline D. Keroack, Yasaman Rezvani, Aditya S. Paul, Keare A. Barazorda, Niel C. Bauer, Jacob A. Tennessen, Samantha A. Sack, Cristina K. Moreira, Marc-Jan Gubbels, Marvin J. Meyers, Kourosh Zarringhalam, Manoj T. Duraisingh

**Affiliations:** 1 Department of Immunology and Infectious Diseases, Harvard T. H. Chan School of Public Health, Harvard University, Boston, MA USA; 2 Division of Emerging and Transfusion Transmitted Diseases, Food and Drug Administration, Silver Spring, MD USA; 3 Department of Mathematics, University of Massachusetts Boston, Boston, MA USA; 4 Department of Biology, Boston College, Chestnut Hill, MA USA; 5 Department of Chemistry, Saint Louis University, Saint Louis, MO USA; 6 Institute for Drug and Biotherapeutic Innovation, Saint Louis University, Saint Louis, MO USA; 7 Center for Personalized Cancer Therapy, University of Massachusetts Boston, Boston, MA USA

**Keywords:** Parasite biology, Parasite genetics, Antiparasitic agents

## Abstract

Egress from host cells is fundamental for the spread of infection by apicomplexan parasites, including *Babesia* species. These tick-borne pathogens represent emerging zoonoses, but treatment options are limited. Here, using microscopy, transcriptomics and chemical genetics, we identified signalling, proteases and gliding motility as key drivers of egress by *Babesia divergens*. We developed reverse genetic tools in *B. divergens* to perform a knockdown screen of putative mediators of egress, identifying kinases and proteases involved in distinct steps of egress (aspartyl protease (ASP) 3 and kinases cGMP-dependent protein kinase (PKG) and calcium-dependent protein kinase (CDPK4)) and invasion (ASP2, ASP3 and PKG) of red blood cells. Inhibition of egress stimulates additional rounds of intracellular replication, indicating that exit from the replication cycle is uncoupled from egress. Chemical genetics validated PKG, CDPK4, ASP2 and ASP3 as druggable targets in *Babesia* spp. and identified promising compounds for babesiosis treatment. Taken together, egress in *B. divergens* more closely resembles egress in *Toxoplasma gondii* than in the more evolutionarily related *Plasmodium* spp.

## Abstract (web summary)

Analysis of *Babesia divergens* egress from host cells reveals druggable targets and identifies compounds for potential babesiosis treatment.

## Main

*Babesia* spp. are tick-borne pathogens that infect, grow in and destroy their host red blood cells (RBCs). Several *Babesia* spp., including *Babesia microti* and *Babesia divergens*, are emerging zoonotic pathogens that can cause fatal disease^1^. *Babesia* spp. cause substantial economic losses globally owing to disease in livestock and companion animals^2^. Current treatment and prevention options suffer from poor efficacy, spontaneous resistance and severe side effects or render the animal products unsuitable for human consumption^1^.

Protozoans in the Apicomplexa phylum, including *Babesia*, are single-celled parasites. The majority of apicomplexan research has been performed in *Plasmodium* and *Toxoplasma gondii*. Knowledge of a wider range of apicomplexan biology will identify conserved essential functions that could be targeted for broad-spectrum anti-apicomplexan drugs. Stable transfection, clustered regularly interspaced short palindromic repeats (CRISPR)–CRISPR-associated protein 9 (Cas9) and inducible knockdown systems have been developed for multiple *Babesia* spp.; however, the genetic tools remain limited compared with *Plasmodium* and *T. gondii*^3^. Substantial effort has been used to develop inhibitors of host cell egress in *Plasmodium* and *T. gondii*^4–6^, with the potential to repurpose drugs for *Babesia*. Egress in *Plasmodium* and *T. gondii* (reviewed in refs. ^7,8^) begins when intrinsic or extrinsic signals activate signalling pathways that result in the release of lytic factors (proteases, phospholipases and perforin-like proteins (PLPs)) from the micronemes and exonemes to allow parasite egress, while the process remains poorly characterized in *Babesia*.

Here we have used cellular, genetic and genomic approaches to define the key cellular features and molecular mediators of *Babesia divergens* egress. We have developed and used stable transfection, a CRISPR–Cas9 system and inducible knockdown systems, combined with small-molecule inhibitors, to determine that the kinases, cyclic guanosine monophosphate (cGMP)-dependent protein kinase (PKG) and calcium-dependent protein kinase (CDPK4), and aspartyl proteases 2 and 3 (ASP2 and ASP3), are required for separate sequential steps in egress and/or invasion of the host RBC. Chemical genetic approaches show that these molecules present validated druggable targets and identify compounds that could be directly repurposed or further developed for babesiosis treatment.

## Results

### An induced-egress assay to study *B. divergens* host cell egress and invasion

**Fig. 1:**
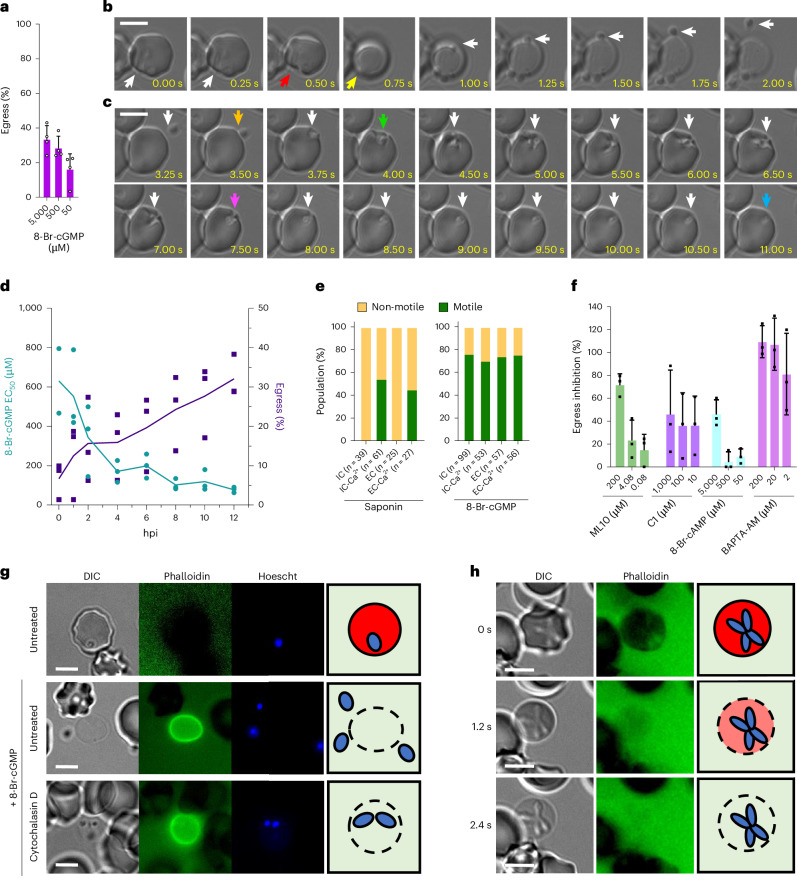
Dynamics of host cell egress and invasion by *B. divergens.* **a**, Percentage of parasites that egress when treated with 8-Br-cGMP. Data are normalized to an RPMI-treated control (0%). **b**,**c**, Time-lapse microscopy of *B. divergens* egress (**b**) and invasion (**c**). Panels **b** and **c** follow the same parasite egressing from one cell and invading a new RBC. Coloured arrows indicate the following: red, initial deformation of the RBC in egress; yellow, ‘rounding-up’ associated with permeabilization of the RBC; orange, initial RBC contact during invasion; green, initial deformation of the RBC during invasion; purple, beginning of internalization; blue, completion of invasion; and white, follows the parasite. **d**, Egress was induced throughout one replication cycle (12 h) of parasites synchronized by merozoite isolation using a range of 8-Br-cGMP concentrations. The half-maximal effective concentration (EC_50_) of 8-Br-cGMP is shown in teal, and the percentage of parasites that egress at 2 mM 8-Br-cGMP is shown in purple. Data are normalized to 0% being a no-8-Br-cGMP-treated control. **e**, Percentage of parasites that become motile when the iRBC is permeabilized by saponin, or induced to egress with 8-Br-cGMP, in the stated buffer. IC and EC refer to intracellular (IC, 140 mM K^+^, 5 mM Na^+^) and extracellular (EC, 5 mM K^+^, 140 mM Na^+^) buffer with or without 2 mM calcium (Ca^2+^). *n* is a single cell visualized by live microscopy and is shown for each condition in the *x* axis. The data are pooled from multiple experiments performed on separate days. **f**, Screen of small molecules for inhibition of 8-Br-cGMP-mediated induced egress. Data are normalized to 100% using an RPMI-only control and 0% using an RPMI-plus-8-Br-cGMP control. **g**, *B. divergens* iRBCs pretreated with cytochalasin D and induced to egress with 8-Br-cGMP. Phalloidin stains the permeabilized RBC. **h**, Time-lapse images showing RBC permeabilization by cytochalasin D*-*treated *B. divergens* during 8-Br-cGMP-induced egress. Phalloidin is excluded from the intact RBC. For **b**, **c** and **e**–**h**, 500 µM 8-Br-cGMP was used. For **a**, **d** and **f**, each point represents the mean of an individual experiment performed in technical triplicate and *n* = 3. For **a** and **f**, the mean ± s.d. of three biological experiments performed in technical triplicate is shown. Scale bars, 5 µm (**b**, **c** and **g**–**h**).

**Extended Data Fig. 1:**
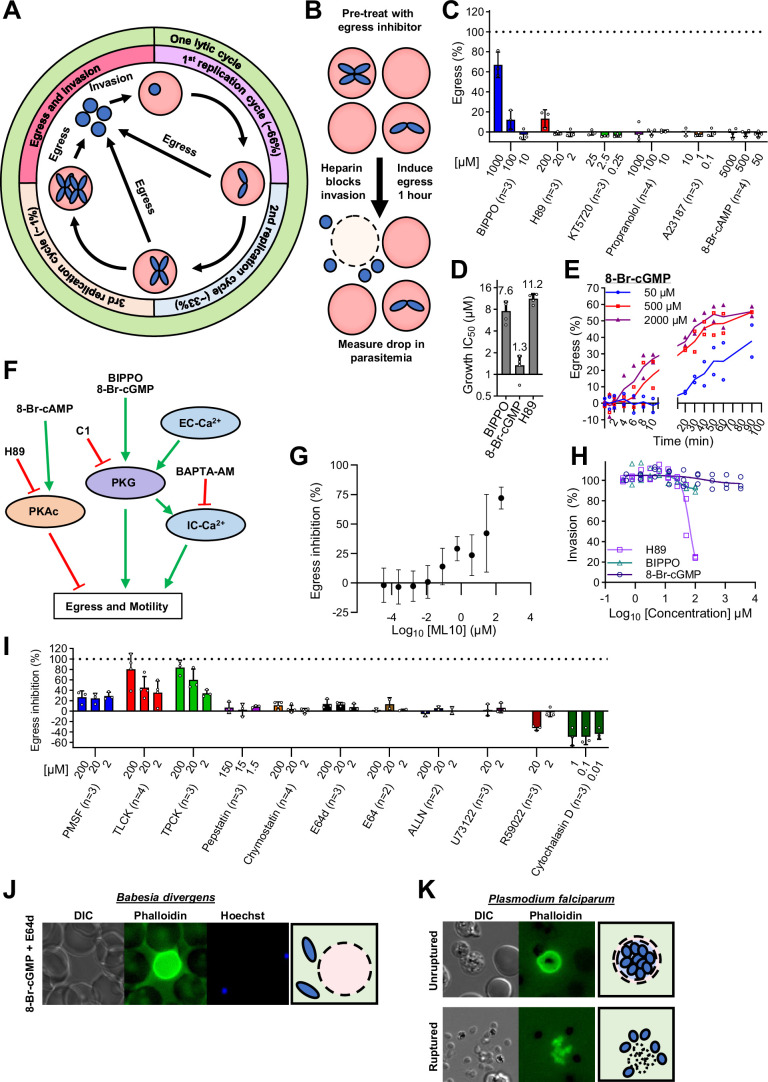
Defining the molecular mediators of egress using small molecule inhibitors. (**A**) Diagram of a lytic cycle of *B. divergens*. In this study, a replication cycle refers to one cell undergoing division to form two (*Babesia* spp. and *T. gondii*) or more (*Plasmodium* spp.) daughter cells. A lytic cycle refers to the time from when a host cell is first invaded to when parasites egress from the same host cell, which can contain multiple replication cycles for *Babesia* spp. and *T. gondii*, but only one for *Plasmodium* spp. The number of replication cycles per lytic cycle is variable. The percentage shown in brackets for the *B. divergens* diagram represent the approximate percentage of parasites that egress after one, two or more replication cycles (derived from Extended Data Fig. 4A). (**B**) Diagram of the flow cytometry-based egress assay. Egress is measured as the reduction in infected cells. (**C**) Percentage of parasites that egress when treated with each compound, as measured by flow cytometry. Data is normalized so the no drug control is 0%. n is stated in the x-axis. (**D**) IC_50_ of each compound against proliferation in a 72 h assay. n = 4. (**E**) Induced egress was measured over time with three concentrations of 8-Br-cGMP. n = 2. (**F**) Model of the egress signaling pathway in *B. divergens* with activators shown with a green arrow, and inhibitors with a red line. (**G**) Dose-response of egress inhibition by ML10. n = 3. (**H**) Invasion inhibition by each compound. Isolated merozoites were pre-incubated with the inhibitor for 10 min before adding RBCs. n = 3 (**I**). Screen for inhibitors of 8-Br-cGMP mediated induced egress. Data is normalized to 100% as a no 8-Br-cGMP control and 0% as RPMI plus 500 µM 8-Br-cGMP. n is stated in the x-axis. For C-I, each point is the mean of a biological replicate performed in technical triplicate. The mean ± SD is shown. (**J**) Pre-treatment with E64d does not alter 8-Br-cGMP induced egress of *B. divergens*. (**K**) *P. falciparum* parasites egressing in the presence of phalloidin, which stains the RBC cytoskeleton. Top. A parasite that has begun egress and has permeabilized the RBC. The merozoites remain in the permeabilized RBC. Bottom. Merozoites that have completed egress. The RBC cytoskeleton has fragmented to release the merozoites.

For controlled studies of egress in *B. divergens*, a flow cytometry-based assay was used to screen for egress induction, which otherwise occurs asynchronously, using known *T. gondii* egress-inducing compounds (Fig. 1a and Extended Data Fig. 1a–c). These compounds, 8-Br-cGMP (cell-permeable and hydrolysis-resistant analogue of cGMP that activates PKG), BIPPO (phosphodiesterase inhibitor) and H89 (cAMP-dependent protein kinase A (PKA) inhibitor), induced egress and inhibited parasite replication (Fig. 1a and Extended Data Fig. 1c–e)^9,10^. Extended Data Fig. 1f shows the putative egress signalling pathway.

To elucidate the cellular features of *B. divergens* egress and invasion, we used video microscopy to follow 8-Br-cGMP-mediated induced egress. As previously observed^11^, *B. divergens* egress is frequently initiated when the intracellular parasite contacts and deforms the host cell (76% (16/21) of egress events; Fig. 1b, red arrow). The RBC then ‘rounds up’ (mean ± s.d. = 2.6 s ± 3 s, *n* = 17) (Fig. 1b, yellow arrow). ‘Rounding’ in *Plasmodium falciparum* occurs preceding parasitophorous vacuole membrane (PVM) rupture, possibly owing to PVM permeabilization^8^. In *B. divergens*, which lack a PVM, RBC permeabilization probably causes rounding. Parasites become motile and escape the permeabilized RBC (mean ± s.d. = 1 s ± 2.4 s, *n* = 28, permeabilization to escape). *B. divergens* egress is rapid (mean ± s.d. = 3.9 s ± 6.0 s, *n* = 15, first motility or RBC deformation to escape) compared with *P. falciparum* egress (~5–10 min)^8^. After contact, the parasite strongly deforms the RBC around itself (known as pre-invasion in *Plasmodium*) (Fig. 1c, green to purple arrows; mean ± s.d. = 6 s ± 2.7 s, *n* = 14), followed by parasite entry with little deformation of the RBC (internalization phase), matching previous observations in *B. divergens* (Fig. 1c, purple to blue arrows, mean ± s.d. = 4.5 s ± 1.1 s, *n* = 10; Supplementary Video 1)^11,12^. The total time from RBC contact to complete internalization is 10.8 s ± 3.0 s (mean ± s.d., *n* = 10).

To test whether *B. divergens* can egress throughout replication, parasites were synchronized to a 20-min window and egress was induced every 1–2 h (Fig. 1d). The increased 8-Br-cGMP sensitivity and number of parasites that egress as the parasites mature suggest it is more strongly primed to egress when fully mature, but can egress throughout replication (Fig. 1d). This resembles *T. gondii* that can be induced to egress throughout replication^13^, whereas *P. falciparum* egress is restricted to the end of the lytic cycle^9,14^.

### Loss of host cell integrity induces *B. divergens* motility and egress

To test whether *B. divergens* motility is induced by exposure to extracellular conditions, we observed parasites after saponin-induced RBC lysis in buffers mimicking intracellular (IC) or extracellular (EC) potassium and sodium concentrations, plus or minus calcium. Parasites in either buffer without Ca^2+^ were nonmotile (Supplementary Video 2), whereas those with Ca^2+^ became motile and could escape the permeabilized RBC (Fig. 1e and Supplementary Video 3). In addition, 8-Br-cGMP bypassed the requirement for extracellular calcium to initiate motility (Fig. 1e). Together, these results suggest that motility is induced by exposure to extracellular concentrations of Ca^2+^, probably acting upstream or in parallel to PKG, but is not regulated by Na^+^ or K^+^. Similarly, *T. gondii* exposure to extracellular albumin, potassium and calcium induces egress^15^. *P. falciparum* senses lipids and potassium although their roles are less clearly defined^16–18^. Extracellular calcium is required for efficient invasion, but not egress, of *P. falciparum*^17,19,20^.

### Chemical inhibition of kinases, proteases and calcium signalling all impair egress

To identify the molecular processes of *B. divergens* egress, we used flow cytometry to screen for compounds, selected from *Plasmodium* or *T. gondii* studies, that inhibit 8-Br-cGMP-induced egress. BAPTA-AM (intracellular calcium chelator) completely blocked egress (Fig. 1f). The apicomplexan PKG inhibitors ML10 and compound 1 (C1) inhibited egress by 72% and 47% at high micromolar to millimolar doses; ML10 showed a dose-dependent response (Fig. 1f and Extended Data Fig. 1g). Furthermore, 8-Br-cAMP (PKA activator) inhibited egress by 46%, which together with the H89-induced egress result suggests a role in PKA regulating egress; however, the significance of these results is unclear given the high concentrations required (Fig. 1f and Extended Data Fig. 1c). H89 strongly inhibits invasion of free merozoites, while BIPPO and 8-Br-cGMP had no effect (Extended Data Fig. 1h). Inhibitors of serine proteases, including TPCK, TLCK and PMSF, reduced induced egress, whereas inhibitors of other protease classes show modest inhibition (<20%) (Extended Data Fig. 1i). The short incubation time (15 min) may not identify inhibitors of pathways that act earlier (for example, PfPMX/TgASP3 orthologues)^21–23^. Inhibitors of lipid signalling that block egress in *T. gondii* and/or *Plasmodium*, including U73122 and propranolol, which target PI-PLC and phosphatidic acid phosphatase, respectively, did not influence egress, whereas the diacylglycerol kinase inhibitor R59022 instead enhanced induced egress (Extended Data Fig. 1i)^8,15^. In agreement with the flow cytometry assay, E64d-treated parasites showed no obvious defect in egress, motility or invasion by video microscopy (Extended Data Fig. 1j). These data show a requirement for cGMP, kinase and calcium (intracellular and extracellular) signalling, and serine proteases for *B. divergens* egress. Unlike in *T. gondii*, A23187-induced calcium release does not induce egress in *B. divergens*, suggesting that calcium release is required, but not sufficient for egress (Extended Data Fig. 1c)^15^. A previous study observed A23187-induced egress in *Babesia bovis*, possibly due to differences between species, host cells or technical reasons (for example, calcium concentration)^24^.

### Motility is required for *B. divergens* merozoites to egress from RBCs but not for RBC permeabilization

RBC deformation during egress coincides with direct parasite contact, indicating that the parasites’ actinomyosin motor may be used to physically disrupt the RBC (Fig. 1b)^11^. Egress was induced in the presence of phalloidin, which selectively stains the cytoskeleton of permeabilized RBCs, and cytochalasin D, which inhibits actin polymerization and gliding motility. By microscopy, cytochalasin D-treated parasites can be induced to permeabilize, but not escape, the RBC or show localized deformation (Fig. 1g,h and Supplementary Video 4). The RBC becomes ruffled, rapidly shows a reduced diameter, becomes round and is infiltrated by phalloidin, showing permeabilization (Fig. 1h and Supplementary Video 4). By flow cytometry, cytochalasin D treatment enhances 8-Br-cGMP-induced egress, probably measuring RBC permeabilization (Extended Data Fig. 1i). *T. gondii*-secreted lytic factors and host calpains damage the host cell, allowing the motile parasite to escape^7^. The mechanisms of RBC membrane lysis in asexual *Plasmodium* remain unclear^8^. PLP1 disruption in *B. bovis* leads to a partial egress defect, but is not strictly required for growth^25^. Phalloidin staining in *B. divergens* shows that the permeabilized RBC remains intact (Fig. 1b,c,g,h)^11^; *P. falciparum*, by contrast, fractures the RBC cytoskeleton (using SERA6 protease), bypassing the requirement for gliding motility (Extended Data Fig. 1k)^8^. We were unable to identify SERA protease nor LCAT/PL phospholipase orthologues in *Babesia* spp.^7,8^. These data argue for a mechanism of host cell lysis in *B. divergens* relying centrally on secretion of lytic factors (for example, PLPs); however, parasite motility could also contribute as shown in *T. gondii*^26^.

### Identification of putative egress, motility and invasion genes through transcriptomic analyses

**Fig. 2:**
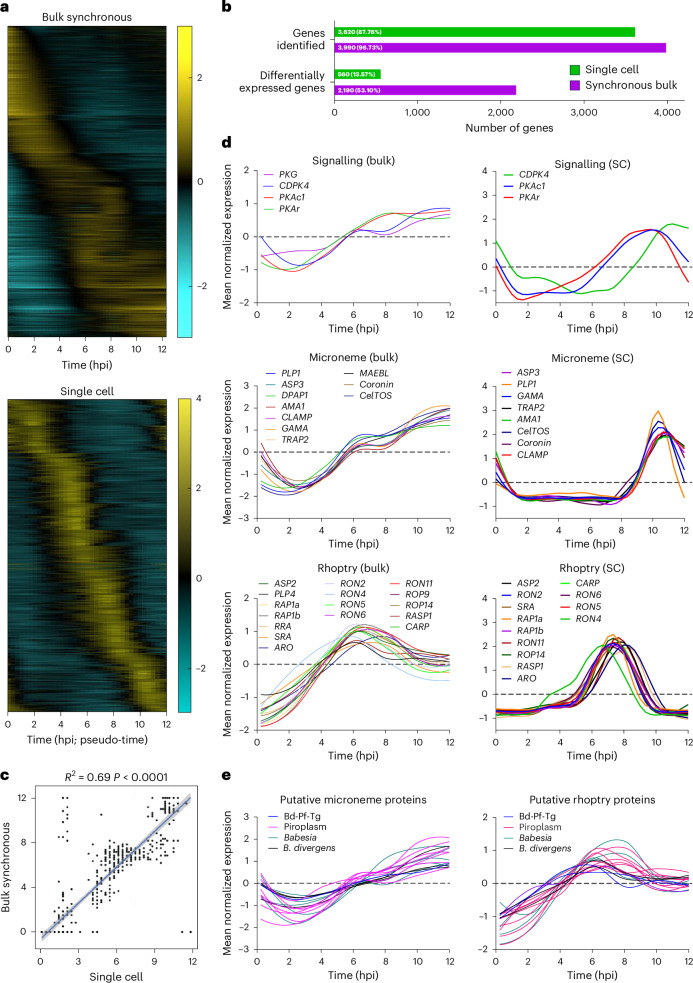
The *B. divergens* transcriptome reveals expression of putative egress and invasion genes. **a**, Expression profile of all differentially expressed genes. **b**, Number of genes identified by both transcriptomic approaches. Differentially expressed genes are defined as having a >1.5-fold change over time for the bulk transcriptome and a ≥2 fold change, adjusted *P* value 0.001, for the single-cell transcriptome (see Methods for details). Green and purple bars represent data from the single-cell and bulk synchronous transcriptomes, respectively. The numbers within the bars show the total number of genes identified and the percentage of genes this represents from all genes. **c**, Correlation of timing of peak expression between the bulk and single-cell transcriptomes. Error shown is the 95% confidence interval (CI) of the fitted linear regression. Statistical analysis was conducted using a two-sided *t*-test for the regression slope. **d**, The expression profiles from bulk synchronous and single-cell data of orthologues of known egress and invasion genes. Some genes are not present in the single-cell analysis owing to lower sensitivity to low-abundance transcripts. **e**, The expression profiles from bulk synchronous data of putative egress and invasion genes. The single cell data were originally generated in ref. ^38^.

**Extended Data Fig. 2:**
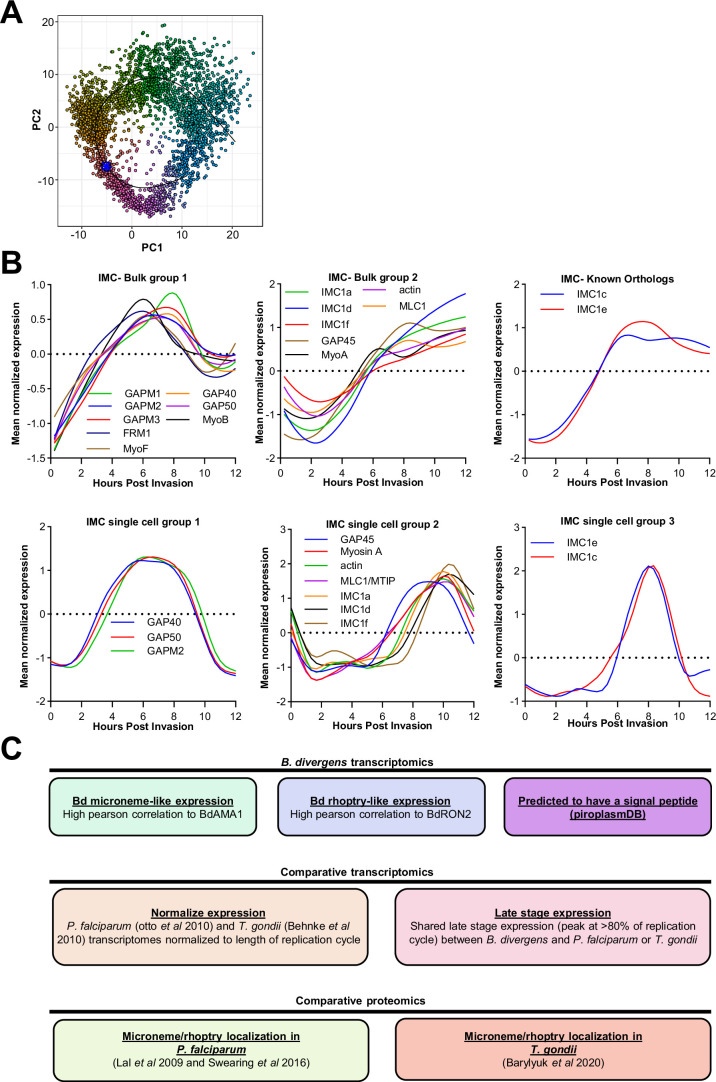
The *B. divergens* transcriptome reveals expression of putative IMC genes and conserved apicomplexan egress and invasion genes. (**A**) PCA plot of the single-cell transcriptomics. (**B**) Expression profiles of IMC proteins fall into three clusters in bulk (top) and single-cell (bottom) transcriptomics. (**C**) An outline of the approaches used to identify novel egress and invasion genes^39,89–91^.

The *B. divergens* lytic cycle has been morphologically defined but remains poorly characterized at the molecular level^27^. Multiple *Babesia* spp. transcriptomes exist at different stages of the life cycle, including replicating parasites compared with free merozoites; however, there are no synchronous time courses that follow replication^3,28–37^. We generated a synchronous bulk transcriptome of one replication cycle (0–12 h). We also used a recent asynchronous single-cell transcriptome where individual gene expression profiles were generated using a pseudo-time analysis by cross-correlation between the bulk and single-cell transcriptomes^38^. Genes with expression changes over time show a transcriptional cascade associated with ‘just-in-time’ gene expression that is observed in *Plasmodium* and *T. gondii* (Fig. 2a and Extended Data Fig. 2a)^39,40^. Fewer genes are included in the single-cell analysis owing to reduced sensitivity (Fig. 2b). The timing of peak expression was well correlated between transcriptomes, supporting the use of the pseudo-time analysis (Fig. 2c). Supplementary Table 1 contains all *B. divergens* gene IDs for this study.

We identified 93 *B. divergens* genes as orthologues or family members of known egress, motility or invasion genes from other apicomplexan parasites. A total of 91 genes were expressed, and 67 showed expression changes over time (Supplementary Table 1). Genes expected to co-localize within the same subcellular compartment (for example, micronemes) based on orthology show similar expression profiles, as observed in *Plasmodium* and *T. gondii* (Fig. 2d and Extended Data Fig. 2b)^39,41^. Signalling proteins, including PKG, PKAc1, PKAr and CDPK4 (PfCDPK4/TgCDPK3), peak ~8–10 h post-invasion (hpi) and remain high until 12 hpi when parasites naturally egress (Fig. 2d). Other putative egress signalling pathway members did not show stage-specific expression (Supplementary Table 1). The cysteine and aspartyl proteases, dipeptidyl aminopeptidase 1 (DPAP1) (no direct orthologue) and ASP3 (PfPMX/TgASP3 orthologue), respectively, show expression profiles matching those of microneme genes (Fig. 2d). The expression of ASP2 (closely related to BdASP3) matches that of rhoptry genes (Fig. 2d). All nine *B. divergens* PLP genes are expressed (Supplementary Table 1). Only PLP1 (TgPLP1/PfPLP3) and PLP4 (no direct orthologue) show expression profiles matching those of microneme and rhoptry proteins, respectively, suggestive of a role in egress and invasion or post-invasion PV breakdown, respectively (Fig. 2d).

Multiple invasion ligands were identified by orthology and show expression profiles matching those of rhoptry or microneme proteins (Fig. 2d). These included TRAP2/P18, RAP1 and AMA1, with demonstrated roles in *Babesia* spp. invasion (Fig. 2d)^42–46^. The orthologues of the rhoptry protein, surface-related antigen (SRA) and microneme proteins, CLAMP, GAMA, MAEBL and CelTOS, represent potential vaccine targets that have not been investigated in *Babesia* spp., with antibodies against CLAMP inhibiting *Theileria equi* growth^47^. Inner membrane complex proteins, involved in cell structure and motility, are transcribed in three groups, warranting future studies (Extended Data Fig. 2b). Many of the genes identified here were differentially expressed between intraerythrocytic and extracellular *B. divergens* parasites^48^, further supporting our data.

A comparative transcriptomic approach was used to identify uncharacterized proteins putatively involved in egress, motility or invasion (methods outlined in Extended Data Fig. 2c). A total of 104 genes were identified, including 31 identified by multiple methods and associated with orthologues with no known function (Fig. 2e and Supplementary Table 1). Of the eight genes found across species, five have a growth phenotype when disrupted in *T. gondii* and *P. falciparum* (Supplementary Table 1).

### A genetic screen reveals the essentiality of PKG, CDPK4, ASP2 and ASP3 for parasite proliferation

We focused on 11 high-priority candidates based on transcriptomics and orthology to apicomplexan egress genes, including the kinases, PKG, PKAc1, PKAc2, CDPK4, CDPK5 and CDPK7; the perforin-like proteins, PLP1 and PLP4; and the proteases, ASP2, ASP3 and DPAP1. PKG has a well-characterized role in egress and invasion in *Plasmodium* and *T. gondii*^49,50^. PKAc1 suppresses egress in *T. gondii* but is required for *P. falciparum* invasion^15,51^. The CDPK family is required for *Plasmodium* and *T. gondii* egress and invasion, although direct orthologues do not always have analogous functions across species^52,53^. PLPs are involved in host cell permeabilization and egress in *T. gondii*, *Plasmodium* sexual stages and *B. bovis*^54^. PfDPAP family members and BdASP2/ASP3 orthologues (PfPMX, PfPMIX and TgASP3) are required for egress and/or invasion in *Plasmodium* or *T. gondii*^21–23,55–57^.

**Fig. 3:**
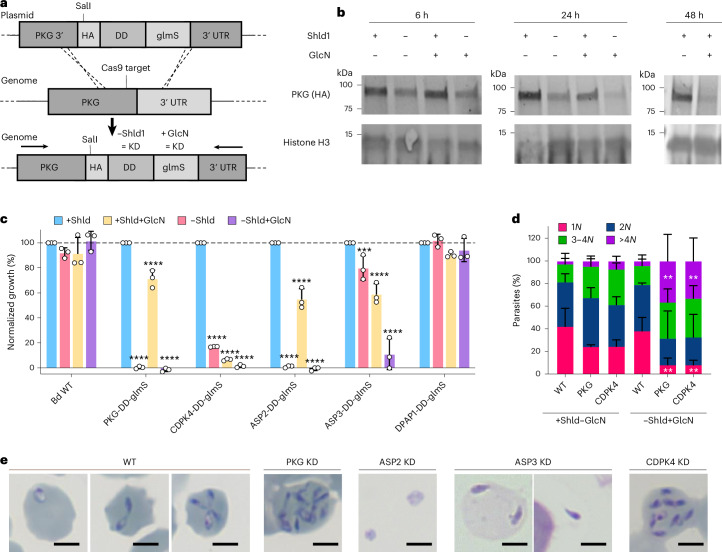
A system for inducible knockdown reveals an essential role for PKG, CDPK4, ASP2 and ASP3 in egress or invasion. **a**, CRISPR–Cas9 is used to introduce an HA-DD-glmS or HA-glmS tag to the 3′ end of *pkg* by homologous recombination. The same approach was used for all genes. The arrows represent primers used to validate integration as shown in Extended Data Fig. 3e. KD, knockdown. **b**, Western blot analysis showing DD and glmS-induced knockdown of PKG over time. **c**, Growth of knockdown parasites over 72 h. Each point represents the mean of an individual experiment performed in technical triplicate and *n* = 3. The mean ± s.d. is shown. Data are normalized so +Shld is 100% for each replicate. Statistical analysis was conducted using two-way ANOVA with Dunnett correction for multiple comparisons (two sided). All statistical values shown are relative to +Shld−GlcN parasites of the same line. ***P = 0.0003; ****P < 0.0001. **d**, The number of parasites per iRBC assessed by microscopy after knockdown (−Shld+GlcN) of PKG or CDPK4 for 40 h. A total of 100 iRBCs were counted per condition, and 3 biological replicates are shown. The mean ± s.d. is shown. Statistical analysis was conducted using two-way ANOVA with Dunnett correction for multiple comparisons (two sided). All statistical values shown are relative to WT +Shld−GlcN parasites with the same *N* compared between conditions. **Exact *P* values are provided for the following in −Shld+GlcN conditions: WT versus PKG 1*N* *P* = 0.0028, WT versus CDPK4 1*N* *P* = 0.0030, WT versus PKG >4*N* *P* = 0.0031, WT versus CDPK4 *P* = 0.0089. **e**, Stained micrographs showing phenotypes after 24 h (ASP2 and ASP3) or 40 h (PKG and CDPK4) of knockdown (KD). Scale bars, 3 µm. For **b**–**e**, +Shld was 500 nM, +GlcN was 1 mM and −Shld−GlcN was 0 nM. Source data

**Extended Data Fig. 3:**
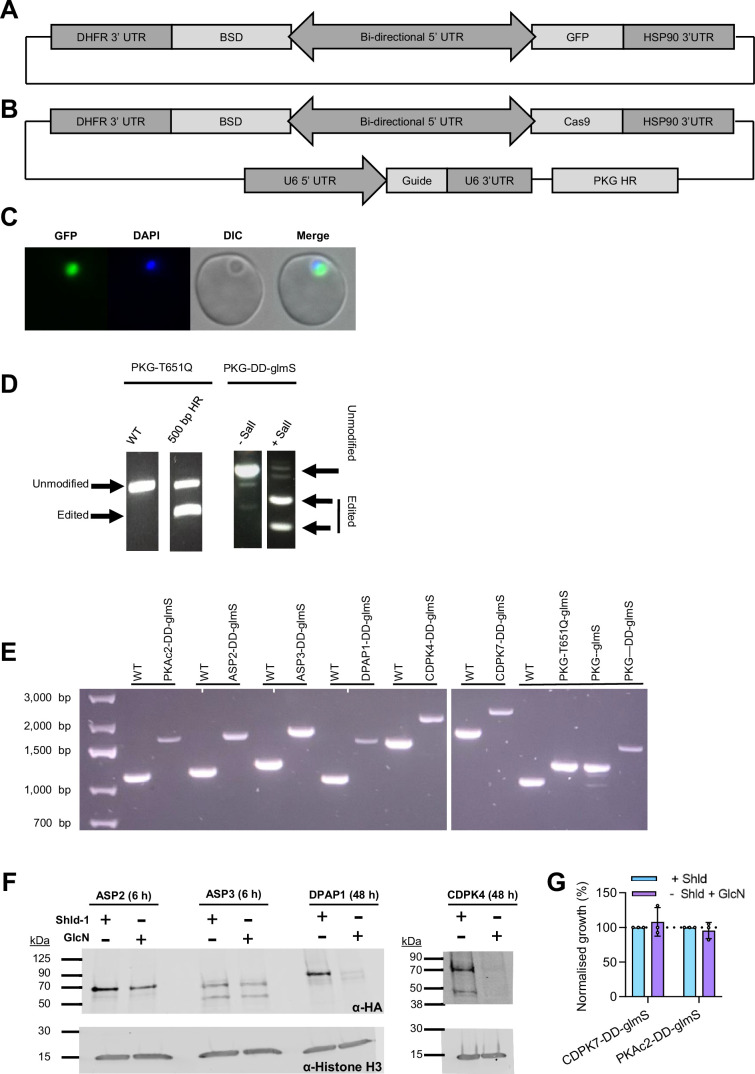
Genetic validation of CRISPR/Cas9 modified parasite lines. (**A**) Schematic of the GFP reporter plasmid. (**B**) Schematic of the CRISPR/Cas9 plasmid with a repair template (PKG HR). Detailed repair template schematics can be found in Figs. 3a and 5a. (**C**) Live cell imaging of *B. divergens* parasites expressing GFP. (**D**) RFLP analysis of the uncloned parasite population after transfection with either a plasmid containing a 500 bp repair template (PKG-T651Q) or with a HA-DD-glmS tag flanked by two 500 bp HR regions targeting PKG (corresponding to diagrams in Figs. 3a and 5a including primer positions). (**E**) PCR and agarose gel electrophoresis to confirm integration of the knockdown tag in each line. The amplified region spans the 3’ tag, with primers outside of the HR (primers are shown in Fig. 3a). The increased size of the PCR product in the transgenic line corresponds to the correctly integrated tag. The expected band sizes for WT and integrated, respectively, for each gene are: PKAc2 (1.1 and 1.6 kb), ASP2 (1.1 and 1.6 kb), ASP3 (1.2 and 1.7 kb), DPAP1 (1.1 and 1.6 kb), CDPK4 (1.6 and 2.1 kb), CDPK7 (1.8 and 2.3 kb), PKG T651Q-glmS/ PKG-glmS (1.1 and 1.3 kb) and PKG-DD-glmS (1.1 and 1.6 kb). (**F**) Western blot analysis showing knockdown by combined DD (0 mM Shld1) and glmS induction (1 mM GlcN). ASP2 and ASP3 knockdown were induced for 6 h due to growth defects after longer periods. DPAP1 and CDPK4 knockdown were induced for 48 h. (**G**) Growth of knockdown parasites over 72 h. Each point represents the mean of an individual experiment performed in technical triplicate and n = 3. The mean ± SD is shown. Data is normalized so +Shld is 100% for each replicate. No statistical differences are observed. Source data

To develop stable transfection, multiple transfection methods and established resistance markers were tested (Extended Data Fig. 3a–c and Supplementary Table 2). Nucleofection of isolated merozoites and blasticidin-S selection was the most efficient method and used for all further transfections. Notably, a recent study selected for stable integration using an hDHFR selection marker in *B. divergens*^58^. A CRISPR–Cas9 system was generated to introduce an hemagglutinin (HA) tag and the *glmS* riboswitch with and without a destabilization domain (DD) inducible knockdown system to the 3′ of each gene (Fig. 3a and Extended Data Fig. 3b)^59,60^. Parasites containing the correct integration reached >1% parasitaemia 12–16 days after transfection for all constructs, except PKAc1, CDPK5, PLP1 and PLP4, which we were unable to tag (Extended Data Fig. 3d,e). Using PKG-HA-DD-glmS parasites, induction of the DD system generated a more rapid (~6 h) and stronger reduction of protein levels (Fig. 3b). Knockdown using glmS was first observable by 24 h but generated strong knockdown by 48 h. Combining both systems resulted in a stronger knockdown than either alone (Fig. 3b). Knockdown was determined using the combined glmS–DD systems at 48 h for CDPK4 and DPAP1, and 6 h for ASP2 and ASP3 (to avoid growth defects). All lines showed knockdown, with ASP2 and ASP3 showing weaker knockdown probably owing to the shorter induction (Extended Data Fig. 3f). We were unable to detect HA-tagged protein for CDPK7 and PKAc1, which may reflect low abundance, partial knockdown or processing of the C-terminus. For PKG, CDPK4, ASP2 and ASP3, double knockdown produced the strongest growth defect, whereas individual systems did not affect proliferation for some genes (Fig. 3c). Induction of knockdown for DPAP1, PKAc2 and CDPK7 lines did not affect proliferation, which could be owing to gene dispensability or insufficient knockdown, and was not further studied (Fig. 3c and Extended Data Fig. 3g).

### PKG and CDPK4 are essential for egress, and their depletion results in additional rounds of intracellular replication

**Extended Data Fig. 4:**
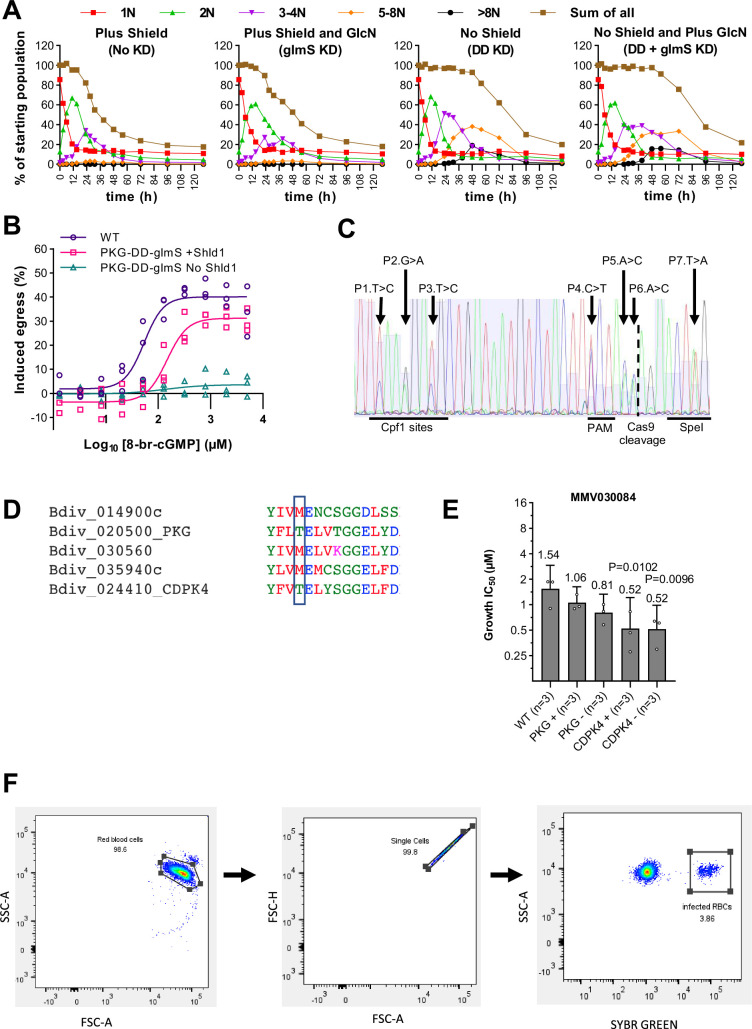
Chemical and genetic targeting of PKG and CDPK4. (**A**) The number of parasites per infected RBC over time with PKG knockdown, assessed by flow cytometry. A single representative experiment is shown of three biological repeats. (**B**) The number of WT or PKG-DD-glmS parasites, with knockdown (no Shld1) or without knockdown (500 nM Shld1) induced for 24 h prior to initiating the experiment, that egress when treated with varying concentrations of 8-Br-cGMP. Each point is the mean of a biological replicate performed in technical triplicate. n = 3. (**C**) Sanger sequencing of pkg in the uncloned parasite population after transfection (T651Q modification). Positions 1-7 are the alternative bases in the homology repair template where editing is expected. (**D**) Alignment of the CDPKs and PKG surrounding the gatekeeper residue (boxed amino acid). (**E**) IC_50_ of MMV030084 for proliferation over 72 h. Each point represents a biological replicate performed in technical triplicate. The mean ± SD is shown. n = 3. Statistical analysis shown is one-way ANOVA with Dunnett correction for multiple comparisons (two-sided). The ‘+’ condition was 500 nM Shld and the ‘-‘ condition was 250 nM Shld. (**F**) A representative flow-cytometry gating strategy to determine the percent of infected RBCs.

**Extended Data Fig. 5:**
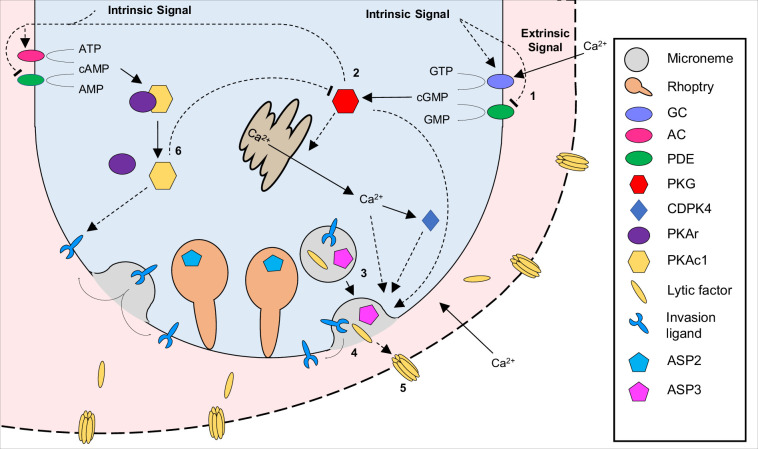
Model of the *Babesia divergens* lytic cycle. 1. An extrinsic signal (calcium) or an intrinsic signal activates guanylate cyclase (GC) and/or inhibits a phosphodiesterase (PDE). 2. The increased cGMP levels activate cGMP-dependent kinase (PKG), which leads to the release of calcium. 3. Calcium, CDPK activity and potentially unknown signals lead to the fusion of micronemes to the parasite plasma membrane. 4. The contents of the micronemes, which have been proteolytically matured by ASP3, are released into the RBC cytosol, including lytic factors, and invasion ligands onto the parasite surface. 5. Lytic factors act to permeabilize the RBC membrane. The permeabilized RBC membrane allows an influx of calcium, acting as a positive feedback loop. 6. Unknown signals, potentially including PKG, lead to the activation of adenylate cyclase (AC) and/or inhibition of a PDE, which increases cAMP levels. The increased cAMP activates PKAc1, which is putatively required for invasion. PKAc1 activity also putatively reduces egress, potentially through inhibition of the PKG signaling pathway. Solid lines represent pathways with a high confidence based on available data for *B. divergens*. Dashed lines indicate processes based primarily on the RNAseq and small molecule inhibitors data from this paper and orthology to *P. falciparum* or *T. gondii*.

We followed replication of PKG and CDPK4 knockdown parasites after synchronized invasion to understand the block in proliferation. Parasites underwent additional rounds of intracellular replication, to form up to 16 parasites per RBC by 40 hpi (Fig. 3d,e and Extended Data Fig. 4a). The fraction of parasites that replicate to >4 per infected RBC (iRBC) is highest in the PKG double-knockdown condition (Extended Data Fig. 4a). PKG knockdown prevented 8-Br-cGMP-induced egress, showing that 8-Br-cGMP acts through PKG (Extended Data Fig. 4b). A schematic of the molecular mediators of egress can be found in Extended Data Fig. 5, and a comparison across apicomplexans in Supplementary Table 3.

### The proteases ASP2 and ASP3 are required for egress and invasion

**Fig. 4:**
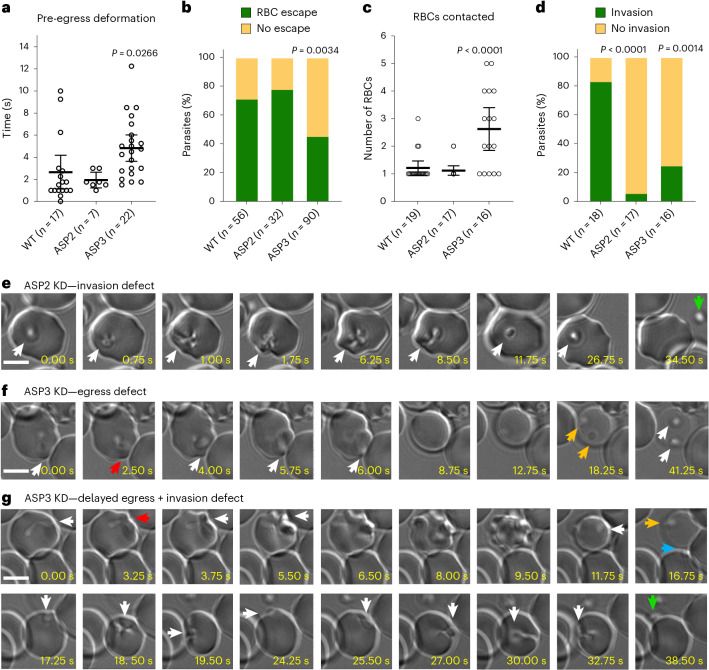
ASP2 and ASP3 are required for egress and/or invasion. **a**, Time taken from initial deformation to RBC lysis during 8-Br-cGMP-mediated parasite egress. The mean ± 95% CI is shown. **b**, Percentage of parasites that escaped the permeabilized RBCs. **c**, Number of RBCs contacted before successful invasion or becoming immotile and failing to invade. The mean ± 95% CI is shown. **d**, Number of parasites that successfully invade after contacting at least one RBC. For **a**–**d**, *n* is a single cell visualized by live microscopy and is shown for each condition in the *x* axis. The data are pooled from multiple experiments performed on separate days. Statistical analysis in **a** and **b** was conducted using one-way ANOVA with Dunnett correction for multiple comparisons (two sided). Statistical analysis in **b** and **d** was conducted using Fisher’s exact test (two sided). Statistics shown are compared with WT parasites. **e**–**g**, Time-lapse microscopy of 8-Br-cGMP-induced egress with knockdown of ASP2 (**e**) and ASP3 (**f**, **g**). Coloured arrows indicate the following: white, follows the parasites; red, initial RBC deformation by the intracellular parasite; orange, failed egress from a permeabilized cell; blue, successful egress; and green, failed invasion and parasite detachment. Scale bars, 5 µm. For **a**–**g**, ASP2 and ASP3 refer to these lines with knockdown (0 mM Shld and 1 mM GlcN). Imaging was performed 18–24 h following induction of knockdown.

The orthologues of *B. divergens* ASP2 and ASP3 in *P. falciparum* (PfPMIX/PMX) and *T. gondii* (ASP3) are part of the protease cascade that matures many microneme and rhoptry proteins required for egress and invasion^21,22^. ASP2 and ASP3 knockdown resulted in an increased number of free merozoites (Fig. 3e). Clusters of parasites were observed in lightly stained RBCs with ASP3 knockdown that probably represent permeabilized RBCs (Fig. 3e). By video microscopy, the time to egress and frequency of escape from lysed RBCs were the same between ASP2 knockdown and wild-type (WT) parasites (Fig. 4a,b). After egress, ASP2 parasites typically bound to, and strongly deformed, a single RBC similar to the WT, but were unable to complete invasion and eventually detached from the cell (Fig. 4c–e and Supplementary Video 5). The time from initial deformation of the RBC by the intracellular ASP3 knockdown parasite to RBC lysis was significantly longer than in WT parasites, and fewer parasites escaped (Fig. 4a,b,f,g). ASP3 knockdown parasites that successfully egressed could bind to and deform the RBC, but maintained gliding motility over the RBC surface and often contacted multiple cells but rarely completed invasion (Fig. 4b–d,f,g and Supplementary Video 6). Together, these results are consistent with the transcriptomic data suggesting that ASP2 functions in the rhoptries and knockdown parasites are able to reorient but not undergo the final step of invasion that requires rhoptry proteins^61–63^. The ASP3 phenotype suggests that it is required for maturation of microneme proteins that are required for RBC lysis and reorientation and/or anchoring of the apical end of the parasite to the RBC before rhoptry release^14,19^. Compared with *T. gondii*, which requires only TgASP3 to process proteins localized to the rhoptries and micronemes, our data suggest that BdASP2 and BdASP3 are functionally orthologous to PfPMIX and PfPMX.

### PKG, CDPK4, ASP2 and ASP3 are druggable targets required for egress and/or invasion

**Fig. 5:**
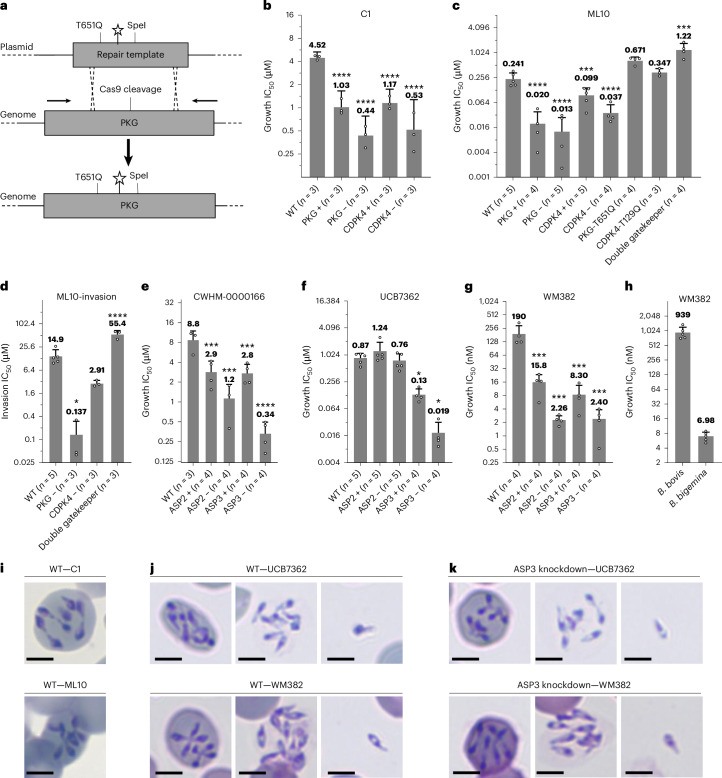
Chemical genetics reveals PKG, CDPK4, ASP2 and ASP3 as druggable targets in *B. divergens.* **a**, CRISPR–Cas9 is used to introduce a putative drug resistance mutation into PKG (T651Q), a silent shield mutation (star) and a silent SpeI restriction site. **b**–**h**, IC_50_ of the stated compound for proliferation (**b**, **c**, **e**–**h**) or invasion of isolated merozoites (**d**). Each dot represents a biological replicate performed in technical triplicate. *n* is given in the *x* axis. The mean ± s.d. is shown. Statistical analysis was conducted using one-way ANOVA with Dunnett correction for multiple comparisons (two sided). Exact *P* values can be found in the source data. *P < 0.05; **P < 0.01; ***P < 0.001; ****P < 0.0001. All statistical values shown are relative to WT parasites. No statistical significance was observed between positive and negative conditions for each parasite line for any drug, which probably reflects partial knockdown induced by genetic modification alone. For growth assays, the positive condition was 500 nM Shld for PKG, CDPK4 and ASP3, and 2 µM for ASP2. For growth, the negative condition was 0 mM Shld for ASP3 and 250 nM for ASP2, PKG and CDPK4. For invasion assays, the negative condition is 350 nM Shld initiated 18 h before the experiment. ‘Double gatekeeper’ refers to the PKG-T651Q/CDPK4-T129Q double mutant. **i**,**j**, WT parasites treated with 20 µM C1 or 5 µM ML10 (**i**) and 10 µM UCB7362 or 300 nM WM382 (**j**) for 48 h. **k**, ASP3 knockdown (no Shld1) parasites were treated with 300 nM WM382 or 10 µM UCB7362 for 48 h. Scale bars, 3 µm. Source data

Substantial effort has been invested to develop specific inhibitors of apicomplexan egress^64–68^. To show that PKG is druggable in *B. divergens*, we used the apicomplexan PKG inhibitor C1 and ML10, a potent and highly specific *P. falciparum* PKG inhibitor with in vivo activity^69,70^. To determine their specificity for BdPKG and BdCDPK4 (the only related kinase with a small gatekeeper residue), we introduced a putative resistance mutation (BdPKG-T651Q and BdCDPK4-T129Q) using CRISPR–Cas9 (Fig. 5a and Extended Data Fig. 4d)^50,71^. A 500-bp repair template was used, achieving ~50% editing, with decreasing efficiency at positions further from the cut site (Extended Data Figs. 3d and 4c). Sequential transfections with the same resistance marker were used to generate double mutants (PKG-T651Q-glmS and PKG-T651Q/CDPK4-T129Q).

The half-maximal inhibitory concentration (IC_50_) of C1 and ML10 were 4.5 µM and 241 nM against WT parasites (Fig. 5b,c). For C1, 10.3- and 8.5-fold increases in potency were observed against PKG and CDPK4 knockdown parasites, respectively (Fig. 5b). For ML10, 18.5- and 6.5-fold increases in potency were observed against PKG and CDPK4 knockdown parasites, respectively (Fig. 5c). Genetic modification alone results in sensitization to inhibitors that probably reflects partial knockdown induced by the DD or glmS tag in the absence of induction, as previously observed in other *Plasmodium* recombinants (Extended Data Fig. 4b)^72,73^. A trending, but not statistically significant, shift in the IC_50_ of ML10 was observed between WT (241 nM) and PKG-T651Q (671 nM) or CDPK4-T129Q parasites (347 nM). The double PKG/CDPK4 gatekeeper line (IC_50_ 1,220 nM) showed a statistically significant 5.1-fold decrease in sensitivity compared with the WT (IC_50_ 241 nM) (Fig. 5c). Together, these results support a model in which ML10 targets both PKG and CDPK4 to inhibit parasite proliferation. Another *P. falciparum* PKG inhibitor with in vivo activity, MMV030084, showed moderate potency (IC_50_ 1.5 µM) and unclear synergy with either kinase (Extended Data Fig. 4e)^74^. The ability to rapidly and specifically inhibit PKG or CDPK4 with ML10 when combined with partial knockdown was used to investigate their roles during RBC invasion. PKG or CDPK4 knockdown increased the sensitivity to ML10 invasion inhibition by 109- and 5.1-fold, respectively; however, only the PKG result was statistically significant (Fig. 5d). The double PKG/CDPK4 gatekeeper line showed a statistically significant 3.7-fold decrease in sensitivity, together suggesting that PKG is required for invasion, while the role of CDPK4 requires further investigation (Fig. 5d).

**Extended Data Fig. 6:**
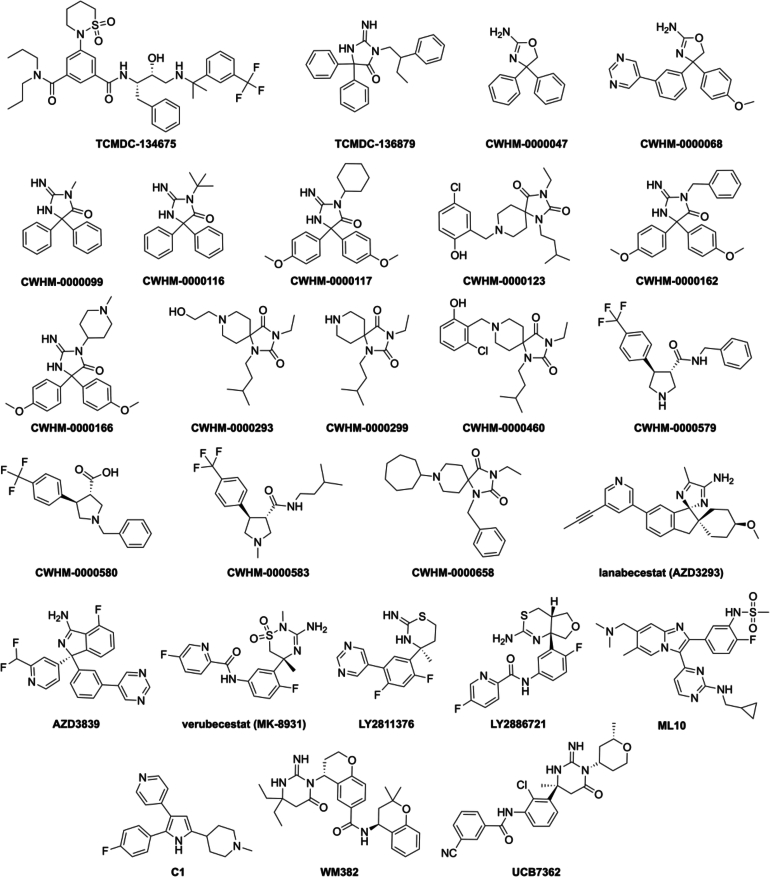
**Structure of all compounds used in this study**.

To determine the druggability of ASP2 and ASP3, we screened 24 aspartyl protease inhibitors, including compounds with known activity against *P. falciparum* PfPMIX and PfPMX, and clinical human beta-secretase inhibitors (Extended Data Fig. 6)^22,75–80^. A total of 16 compounds showed an IC_50_ < 25 µM, including 3 with sub-micromolar activity, WM382 (IC_50_ 190 nM), TCMDC-134675 (IC_50_ 891 nM) and UCB7362 (IC_50_ 873 nM) (Fig. 5e–g and Table 1)^22,78–80^. Multiple compounds were synergistic with ASP2 and/or ASP3 knockdown (Table 1). WM382 and CWHM-0000166 (IC_50_ 8.8 µM) were synergistic with ASP2 (84- and 7-fold, respectively) and ASP3 (79- and 26-fold, respectively) inhibition (Fig. 5e,g). UCB7362 was synergistic with only ASP3 knockdown (46-fold) (Fig. 5f). Concentrations of WM382 that inhibit *B. divergens* growth have been observed in a rodent model, and a WM382 analogue, MK-7602, has entered clinical trials (NCT06294912)^6^, suggesting that it could be repurposed directly for babesiosis treatment^80^. We also tested WM382 against the two most important agricultural *Babesia* spp., *B. bovis* and *Babesia bigemina*, which showed an IC_50_ of 939 nM and 6.7 nM, respectively (Fig. 5h).

**Table 1:** IC_50_ values of aspartyl protease inhibitors against *B. divergens*

	Wild-type IC_50_ (±s.d.) (µM)	ASP2 (high Shld1) IC_50_ (±s.d.) (µM)	ASP2 (low Shld1 = knockdown) IC_50_ (±s.d.) (µM)	ASP3 (high Shld1) IC_50_ (±s.d.) (µM)	ASP3 (low Shld1 = knockdown) IC_50_ (±s.d.) (µM)
CWHM-0000032	0.891 (±0.307)	0.320 (±0.212)	0.478 (±0.288)	0.362 (±0.129)	0.246 (±0.095)
CWHM-0000033	4.03 (±1.90)	1.57 (±0.17)	1.63 (±0.94)	1.39 (±0.26)	0.539 (±0.358)
CWHM-0000099	>25	11.8 (±0.8)	3.99 (±3.15)	16.3 (±3.7)	2.85 (±1.52)
CWHM-0000117	6.81 (±3.08)	2.66 (±0.55)	2.56 (±1.32)	3.17 (±0.61)	1.13 (±0.50)
CWHM-0000166	8.80 (±3.18)	2.89 (±1.27)	1.15 (±0.70)	2.76 (±0.96)	0.337 (±0.157)
CWHM-0000047	19.5 (±5.3)	18.5 (±12.9)	6.32 (±5.84)	16.9 (±9.0)	8.47 (±5.35)
CWHM-0000579	12.6 (±6.6)	7.49 (±4.73)	11.1 (±6.5)	9.12 (±6.12)	7.32 (±6.48)
CWHM-0000162	13.3 (±9.4)	7.15 (±6.45)	6.47 (±5.35)	8.00 (±5.06)	6.41 (±6.74)
SLU-0010620	19.8 (±5.3)	10.1 (±3.4)	4.67 (±3.06)	14.1 (±2.6)	10.0 (±5.4)
SLU-0010622	10.9 (±3.3)	6.38 (±3.27)	1.89 (±2.78)	8.04 (±3.36)	5.49 (±4.29)
CWHM-0000068	16.6 (±7.6)	15.7 (±12.6)	8.02 (±0.89)	13.3 (±2.9)	9.88 (±4.84)
CWHM-0000116	>25	>25	18.8 (±8.2)	20.3 (±11.3)	21.4 (±10.2)
CWHM-0000123	9.46 (±3.66)	8.39 (±4.39)	10.8 (±2.8)	11.2 (±8.7)	9.22 (±1.76)
CWHM-0000293	>25	>25	>25	>25	>25
CWHM-0000299	>25	>25	>25	>25	>25
CWHM-0000460	>25	12.3 (±10.2)	12.2 (±1.5)	12.4 (±5.6)	12.6 (±4.0)
CWHM-0000580	>25	>25	>25	>25	>25
CWHM-0000583	>25	14.2 (±9.5)	14.5 (±8.9)	13.8 (±5.1)	14.1 (±6.9)
CWHM-0000658	16.2 (±9.8)	12.6 (±11.9)	6.47 (±3.82)	9.35 (±3.77)	7.82 (±5.45)
SLU-0010621	25.7 (±12.3)	15.8 (±9.6)	8.64 (±4.98)	15.8 (±3.6)	18.6 (±13.4)
SLU-0010623	24.2 (±10.7)	6.37 (±3.46)	2.57 (±2.67)	13.4 (±7.2)	12.1 (±8.2)
SLU-0010624	18.5 (±5.7)	6.38 (±3.72)	5.76 (±6.77)	9.62 (±4.22)	9.30 (±6.24)
UCB7362	0.908 (±0.232)	1.21 (±0.76)	0.764 (±0.306)	0.113 (±0.021)	0.020 (±0.012)
WM382	0.0341 (±0.0064)	0.00775 (±0.0019)	0.00267 (±0.0014)	0.00695 (±0.0010)	0.00088 (±0.0005)
ML10	0.864 (±0.319)	0.794 (±0.072)	1.56 (±1.24)	0.682 (±0.256)	0.628 (±0.219)
Atovaquone	0.021 (±0.007)	0.020 (±0.005)	0.020 (±0.008)	0.021 (±0.006)	0.025 (±0.011)

Activity of aspartyl protease inhibitors against WT and ASP2 and ASP3 knockdown parasite growth.

The specificity of the compounds was further investigated by microscopy. Treatment of WT parasites with C1 or ML10 resulted in additional rounds of intracellular replication consistent with PKG or CDPK4 knockdown phenotypes (Fig. 5i). Treatment with WM382 or UCB7362 resulted in a mixed phenotype including additional rounds of intracellular replication, clusters of >4 parasites contained in permeabilized RBCs and free merozoites (Fig. 5j). This is similar to ASP3 knockdown, with the added phenotype of additional rounds of intracellular replication. By contrast to PKG or CDPK4 inhibition, ASP3-inhibited parasites typically lyse the RBC after multiple rounds of replication (Fig. 5j). In *P. falciparum*, complete PMX inhibition prevents PVM lysis, while incomplete suppression allows for PVM rupture^21,80^. To determine whether the observed phenotypes were incomplete, we combined ASP3 knockdown with WM382 or UCB7362 treatment at ~125× and ~525× the IC_50_, respectively, and observed the same phenotype as drug alone (Fig. 5k). The lack of additional rounds of replication with knockdown alone probably represents an incomplete phenotype (Fig. 3e). The additional rounds of replication seen following inhibition of ASP3, CDPK4 and PKG suggest that failure to egress by multiple mechanisms results in a default for continuation of the intracellular lytic cycle, but with differences in the ability to eventually lyse the host cell. No synergy was observed between ASP2 or ASP3 knockdown and ML10, suggesting that the kinase and protease egress pathways act independently (Table 1). Together, these data show that PKG, CDPK4, ASP2 and ASP3 are essential and druggable proteins in *B. divergens* that are required for egress and/or invasion. WM382 shows the potential for direct repurposing or further development for the treatment of multiple *Babesia* spp.

## Discussion

Here we establish *B. divergens* as a genetically tractable in vitro model to study essential *Babesia* cell biology. Although transfection systems exist for other apicomplexans^3,81^, the range of tools and resources remains limited outside of *Plasmodium* and *T. gondii*. Egress is similar between *Plasmodium* and *T. gondii*, but with notable differences in their cell biology (for example, division mechanisms) and the host cell niche that place unique pressures on the parasite (for example, nucleated versus enucleated). While the signalling and molecular mechanisms differ in some aspects, we found that *B. divergens* egress at the cellular level closely resembles that of *T. gondii* and has several notable differences to *Plasmodium* despite a more recent common ancestor and sharing the same RBC niche. Similar to *T. gondii*, the *B. divergens* actinomyosin motor is required to allow parasites to pass through the intact RBC cytoskeleton, in contrast to *Plasmodium* that fractures the RBC cytoskeleton to release parasites. *B. divergens* motility is induced by exposure to extracellular calcium that acts as a signal of RBC damage (Fig. 1e and Supplementary Video 3). Previous studies have suggested that *B. divergens* responds to stress (high parasitaemia or drugs) to alter the parasite population structure^27,82^. Similarly, *T. gondii* can sense signals of parasite density and host cell damage to induce egress^7,15^. While *Plasmodium* can sense its environment, its role in egress is unclear and the egress signal is likely to be linked to the cell cycle as it can egress only at the end of replication^16,17^.

In *T. gondii* and *Plasmodium*, the initial egress signals converge to raise cGMP levels and activate PKG. PKG is central to the *B. divergens* egress signalling pathway and is necessary and sufficient to induce egress; however, the role of downstream lipid signalling remains unclear^7,8,15^ (Fig. 1a,f and Extended Data Fig. 4b). Chemical perturbation of BdPKA suggests that it suppresses egress and promotes invasion, differing from *T. gondii* (suppresses premature egress) and *P. falciparum* (required for invasion but not egress)^15,51^ (Fig. 1f and Extended Data Fig. 1c,h). Only BdPKAc1 shows stage-specific expression; however, BdPKAc2 could be involved in these phenotypes (Fig. 2c and Table 1). Of the three CDPKs expressed in *B. divergens*, which act downstream of PKG and calcium release in *Plasmodium* and *T. gondii*, CDPK4 showed late-stage expression and an egress defect when knocked down (Fig. 3e). Inhibition of the egress signalling pathway in *B. divergens* or *B. bovis* (PKG and CDPK4 inhibition or calcium chelation) results in additional rounds of intracellular replication (Fig. 3d,e)^82,83^. In *P. falciparum*, PMIX and PMX proteolytically mature rhoptry and microneme proteins, respectively^22^, whereas *T. gondii* ASP3 performs this function in a post-Golgi compartment^21,23^. The putative subcellular compartmentalization of BdASP2 and BdASP3 based on transcriptomics and their knockdown phenotypes suggest that they are functionally orthologous to PfPMIX and PfPMX, respectively. We hypothesize that the ASP3 inhibition phenotype is due to a block in maturation of lytic factors in *B. divergens*, as is observed in *T. gondii* (for example, PLP1)^26,50^. This implies that *T. gondii* and *Babesia* spp. lack a checkpoint to exit the cell cycle and that the default pathway is to continue replication.

Through knockdown and the introduction of resistance mutations, we identified dual inhibitors of PKG and CDPK4, ASP2 and ASP3, or individual inhibitors of ASP3 (refs. ^22,75,76^; Fig. 5b–d). The dual activity against these proteins may reduce resistance development. WM382 and ML10 are the most potent among the antimalarials tested and show the greatest promise for further development or direct repurposing to treat babesiosis (Fig. 5c–h). ML10 is similarly potent against *B. divergens* (IC_50_ 241 nM) and *B. bovis*^84^ (70 nM), whereas WM382 showed larger differences between *B. divergens* (190 nM), *B. bovis* (939 nM) and *B. bigemina* (7 nM) (Fig. 5c,g,h). While the compounds are less potent than in *P. falciparum* (WM382 IC_50_ 0.6 nM and ML10 IC_50_ 2.1 nM)^70,80^, this is also true for atovaquone (21 nM versus 0.38 nM) (Table 1), which has successfully been repurposed as the frontline human babesiosis treatment. Substantial resources are being used to develop new inhibitors against these targets for malaria, providing a clear path to identify more potent inhibitors against *Babesia*, or for direct repurposing of the next generation of antimalarials.

Here we have established a molecular framework for *Babesia* egress. Several major questions remain, such as how they selectively lyse the PVM after invasion, the role of lipid signalling, the initial egress signal and how they regulate egress to occur after one or more replication cycles. With the cellular, transcriptomic and genetic tools developed here, future studies will be able to answer these questions and reveal the unique biology of *Babesia* as well as conserved processes throughout Apicomplexa, providing a rational basis for the development of therapeutic interventions.

## Methods

### Experimental model and subject details

#### Parasite strains

*B. divergens* strain Rouen 1987, provided by K. Deitsch and L. Kirkman (Weill Cornell Medical College), was cloned by limiting dilution (BdC9) and was maintained in purified Caucasian male O+ human RBCs (Research Blood Components) at 2–4% haematocrit. *B. bovis* strain MO7 and *Babesia bigemina* JG29 were provided by D. Allred (University of Florida) and maintained in purified bovine RBCs (Lampire Biological Laboratories). Parasites were cultured in RPMI-1640 medium supplemented with 25 mM HEPES, 11.50 mg l^−1^ hypoxanthine, 2.42 mM sodium bicarbonate and 4.31 mg ml^−1^ AlbuMAX II (Invitrogen), at 37 °C in a 1% oxygen, 5% carbon dioxide and 94% nitrogen environment.

### Method details

#### Induced egress

To measure egress, mixed-stage parasites at 2% final HCT and 10–15% parasitaemia in a total volume of 40 µl in the presence of the stated drug were incubated at 37 °C for 1 h in a 96-well plate. Where not otherwise stated, 8-Br-cGMP was used at a final concentration of 500 µM. For inhibition of induced egress, parasites were first incubated with the stated compound at 1.33× concentration for 15 min at 37 °C, before induced egress with the addition of 8-Br-cGMP to a final concentration of 500 µM and 1× inhibitor concentration. A final concentration of 100 µg ml^−1^ of heparin was added to prevent reinvasion. After 1 h, the parasites were washed 3× with PBS and stained with 1:5,000 SYBR Green I. Parasitaemia was determined by flow cytometry (Macs Quant, Miltenyi) and analysed in FloJo. Induced egress was calculated as the drop in parasitaemia of the treated parasites as a percentage of the RPMI-only sample.

#### Live microscopy

All videos were taken on a Zeiss Axio Observer using a 60× oil immersion lens inside a chamber heated to 37 °C. Before imaging, parasites were allowed to settle at the bottom of a glass-bottom slide (Ibidi, catalogue number 80827/81817) at 37 °C in a 5% CO_2_ incubator for 10–15 min. Small-molecule inhibitors were included in this incubation as necessary. Immediately before the imaging, the medium was removed and replaced with the RPMI/IC/EC with 500 µM 8-Br-cGMP, and any small-molecule inhibitors being tested. For IC/EC experiments, the final buffer contained 0.0075% (w/v) saponin (Calbiochem, catalogue number 558255) to lyse the RBC. Alexa Fluor 488 Phalloidin (Invitrogen, catalogue number A12379) was added to RPMI at a concentration of 1/150 where stated. Microscopy was performed at 4 frames per second for all experiments, except those that included fluorescent phalloidin, which were performed at 1 frame per 1.2 s. All images were taken within 20 min of removal from the 5% CO_2_ incubator. *P. falciparum* mature schizonts were isolated by magnetic affinity purification (MACs LS column, Miltenyi) and imaged as per the *B. divergens* protocol. Images were processed in Zen 2 (Zeiss) and ImageJ/Fiji.

#### Isolation of free merozoites for invasion assays, synchronization and transfection

Approximately 1–2 ml of packed iRBCs at 20–30% parasitaemia was used to isolate free merozoites using a modified protocol from ref. ^27^. Briefly, the iRBC was resuspended to 10% HCT in RPMI and passed through two 1.2-µM filters. The isolated merozoites and RBC debris were pelleted at 3,000 × *g* for 3 min, and the supernatant was removed. It is important to perform the remaining steps as soon as possible to minimize a drop in parasite viability. For invasion assays, the merozoites were resuspended in RPMI and added to a 96-well u-bottom plate containing 1.33× final drug concentration (30 µl total) and incubated at 37 °C for 10 min. Fresh RBCs were then added to a final concentration of 2% HCT and 1× drug concentration (40 µl total). The plate was then incubated at 37 °C with shaking at 600 rpm for 20 min. The assay was stopped by washing the parasites three times with 200 µl PBS, 400 × *g* for 2 min, or by adding 200 µl 4% paraformaldehyde. Parasitaemia was determined by flow cytometry as per Induced Egress section above. For synchronization, isolated merozoites were resuspended to a final volume of 1 ml of 20% HCT RBCs in RPMI and allowed to invade for 20 min shaking at 600 rpm and 37 °C. Parasites were then washed 3× with 10 ml RPMI at 400 × *g* to remove free merozoites and cell debris. Subsequently, 100 µg ml^−1^ heparin was added to prevent reinvasion throughout the time course when stated in the figure legend.

#### Plasmid construction

The sequences of all primers and synthesis products used in this study are found in Supplementary Table 4. The bidirectional promoter between Bdiv_030590 and Bdiv_030580c was amplified using primers BE-8 and BE-9, and cloned into XhoI/BamHI sites of pPfEF-GFP-BSD (Bdiv_030590 side drives GFP/Cas9 expression). The DHFR (Bdiv_030660, primers BE-21/22) and HSP90 (Bdiv_037120c, primers BE-32/33) 3ʹ UTRs were cloned into EcoRI/HindIII and SpeI/NotI sites, respectively, for the selection marker or GFP/Cas9, respectively. Cas9 was amplified from pDC2–Cas9 using primers BE-124/125 and cloned into the XhoI/SpeI sites in place of GFP. The U6 promoter, bbs1 sites (for the guide), guide tracer and scaffold, U6 terminator and PKG-T651Q repair template were synthesized by integrated DNA technologies (IDT) (synthesis 1) and cloned into the EcoRI site by Gibson assembly to make the pEF-Cas9-PKG-T651Q plasmid. For the single-guide RNA (sgRNA), oligos were either phosphorylated, annealed or cloned into the BbsI sites, or the sgRNA was amplified using the corresponding ‘guide PCR’ primer (for example, BE-551) and BE-550 (universal for all). For all inducible knockdown lines, the HA-glmS or HA-glmS-DD tags were amplified using primers BE-536/537. The 5′HR (HR1, 3′ end of the gene) and 3′HR (HR2, 3′ UTR of gene) were amplified using the corresponding HR1 and HR2 primers from Supplementary Table 4 for each gene (for example, BE_512-515 for CDPK4). The sgRNA, HR1, DD-glmS and HR2 PCR fragments were cloned into the Bbs1/PacI sites of the pEF-Cas9-PKG-T651Q plasmid in a single Gibson reaction. Correct integration of plasmids was confirmed with primers from Supplementary Table 4 labelled ‘test integration’ (for example, BE_610/611 for CDPK4).

#### Transfection

For transfection of iRBCs using the Biorad Genepulser II, 100 µg of DNA in 30 µl combined with 370 µl of cytomix (120 mM KCl, 0.15 M CaCl_2_, 2 mM EGTA, 5 mM MgCl_2_, 10 mM K_2_HPO_4_–KH_2_PO_4_, 25 mM HEPES, pH 7.6) and 200 µl iRBCs (~15% parasitaemia). Transfection was carried out with a Biorad genepulser II set to 0.31 kV and 950 µF. For Amaxa nucleofection of free merozoites, merozoites were isolated from ~3 × 10^9^ iRBCs and all supernatant was removed. It is important to perform the remaining steps as soon as possible to minimize a drop in parasite viability. The pellet, containing free merozoites and cell debris, was resuspended in 100 µl of P3 solution (Lonza) plus 10 µl of water containing 2–10 µg of DNA. For Amaxa nucleofection of intact iRBCs, 10 µl of iRBCs at 10% parasitaemia was resuspended in 110 µl P3 + DNA solution, as per above. Transfection of either free merozoites or iRBCs was carried out in a 4D-Nucleofector System (Lonza), using the FP158 program. After electroporation of free merozoites, the parasite and buffer mixture was immediately transferred to 1 ml of RPMI containing 200 µl packed RBCs and pre-heated to 37 °C. Parasites were allowed to invade at 37 °C with shaking at 600 rpm for 20 min before being washed with 10 ml RPMI to remove the P3 solution and returned to culture. Electroporated intact iRBCs were washed 1× with 10 ml RPMI and returned to culture. Cultures were selected with 15–20 µg of Blasticidin-S.

#### Drug sensitivity and proliferation assays

For all proliferation assays, parasites were diluted to 0.2% parasitaemia in 2% haematocrit (100 µl total) in a 96-well U-bottom plate. *B. divergens* were cultured for 72 h. Parasitaemia was measured by flow cytometry with SYBR Green I (1:5,000). A representative gating strategy is shown in Extended Data Fig. 4f. Testing of kinase and aspartyl protease inhibitors (Fig. 5b–h and Table 1) was performed using a whole-well SYBR assay^85^. C1, ML10, UCB7362 and WM382 were tested in 96-well format, with initial parasitaemia set to 0.25–0.3%, 2% HCT and 100 µl per well, and all other compounds were tested in 384-well format, with initial parasitaemia set to 0.4%, 2% HCT, 40 µl per well and 72 h of proliferation in the presence of the stated compounds. *B. bovis* and *B. bigemina* were tested in 96-well format, with initial parasitaemia set to 0.8–1%, 2% HCT and 100 µl per well and cultured for 48 h. SYBR lysis buffer contained 0.16% saponin, 20 or 50 mM Tris–HCl, 5 mM EDTA and 1.6% Triton X 100; pH was adjusted to 7.4, with 1:1,000 or 1:5,000 SYBR Green I added immediately before use for *B. divergens*, and *B. bovis* and *B. bigemina*, respectively. Where not otherwise stated, +Shld was 500 nM of Shield-1 (Shld1), +GlcN was 1 mM of glucosamine and −Shld−GlcN was 0 nM. The chemical structures of compounds used in this study can be found in Extended Data Fig. 6.

#### RNA-sequencing analysis

Parasites were synchronized by isolating merozoites and allowing invasion to a 20-min window. Parasites were collected at 0, 2, 4, 6, 8, 10 and 12 hpi, as well as the asynchronous starting parasites before merozoite isolation. Two biological replicates were performed (BE and CK samples in SRA), each in technical duplicate (BE-1 and BE-2 in SRA) for each time point. RNA was isolated from parasites using a hybrid protocol of organic extraction combined with column purification. Briefly, parasite pellets were resuspended in 500 µl TRIzol and then extracted with chloroform. The aqueous layer was then purified using Qiagen RNeasy Mini spin columns following the manufacturer’s protocols. RNA was quantified and normalized into individual wells, and libraries were prepared following the Smart-seq2 protocol^86^. Libraries were sequenced on the Illumina platform. For the bulk synchronous RNA-sequencing (RNA-seq) analysis workflow, the quality of reads was assessed using FastQC (version 0.10.1). The reads were trimmed with Cutadapt from the Trim Galore package (version 0.3.7; http://www.bioinformatics.babraham.ac.uk/projects/trim_galore/). The trimmed reads were mapped against the Bdivergens1802A reference genome (PiroplasmaDB release 46) and assembled with HISAT2 (version 2.0.5 released; https://www.ncbi.nlm.nih.gov/pmc/articles/PMC4655817/). SAM files obtained from alignment results were processed using SAMtools (version 1.4.1), and the relative abundances of transcripts were estimated using featureCounts (https://academic.oup.com/bioinformatics/article/30/7/923/232889). For normalization and noise removal, counts per million (cpm) values per gene were calculated using cpm() from the edgeR R package (version 3.24.3; https://www.bioconductor.org/packages/release/bioc/vignettes/edgeR/inst/doc/edgeRUsersGuide.pdf). Genes with cpm values > 2 in at least 3 samples were maintained for further analysis. Gene counts were normalized and scaled to logarithmic form using the trimmed mean of *M* values (TMM) method of edgeR, with DGEList(), calcNormFactors() and cpm() functions. The cpm() parameters were as follows: y=DGEList.obj, log=TRUE, prior.count=3, normalized.lib.sizes=TRUE. Batch effect samples were identified through the analysis of hierarchical clustering and dissimilarities method using the R function hclust() with default parameters and logCPM expression values. These samples were excluded from further analysis. The bad samples were the ones clustered in single batches and were not similar to the rest of the samples based on Euclidean distance metric. For principal component analysis (PCA), PCA was run on genes with fold-change (FC) > 1.5 with prcomp() from R. The first two principal components were chosen for visualization. For orthologue analysis, orthologous genes between *B. divergens* 1802A, *P. falciparum* 3d7, *T. gondii* ME49 and *P. berghei* ANKA (all from VEuPathDB release 46) were assembled using blast (version 2.10.0; https://academic.oup.com/nar/article/36/suppl_2/W5/2505810) and the R package Orthologr (version ‘0.4.0’) with the implemented best reciprocal hit method. For smoothed expression curve analysis, only genes with an average of ≥10 cpm across each time point were maintained. Curves were generated using the loess.smooth function in R (span = 0.5, degree = 2, family = c(‘gaussian’)). Differentially expressed genes were those that showed a ≥1.5-fold change over time. *P. falciparum* data were from ref. ^87^ (downloaded from PlasmoDB). Genes with fragments per kilobase million (FPKM) ≥ 5 were maintained. *T. gondii* data were from ref. ^39^ (downloaded from ToxoDB). *T. gondii* data were manipulated to change the time points to match the other datasets (that is, the starting time point is 6 h from the paper, which is straight after invasion). Pearson correlation was calculated in R using the smoothed curves for AMA1 and RON2. Genes were considered to be highly correlated if they showed ≥2-fold change over time and had a Pearson correlation value of ≥0.9.

#### scRNA-seq workflow

Single-cell RNA-sequencing (scRNA-seq) data were processed using the 10x cell-ranger pipeline and aligned to the *B. divergens* 1802A genome. Counts were subsequently normalized and processed using the R Seurat package. A total of 9,450 cells and 3,620 genes were retained after removing cells and features with low counts (Seurat parameters: min.cells = 10, min.features = 100, nFeature_RNA > 200 nFeature_RNA < 1200). Dimensionality reduction and clustering analysis were performed using PCA and graph-based *K*-nearest neighbors (KNN) as implemented in the Seurat Package. A total of four clusters were identified by the KNN algorithm. To make the size of the data manageable, each cluster was downsampled to include 800 cells. Global differential expression performed on each cluster (log(FC) > 1 and adjusted *P* < 0.01) identified 544 differentially expressed genes. Pseudo-time analysis was performed on the first two PCA components by fitting a principal curve to the data and orthogonally projecting the cells on the curve. Gene expression curves were then constructed using the pseudo-time, and the start of gene expression was identified by cross-correlation analysis between the bulk and single-cell expression curves. Further details can be found in ref. ^38^.

### Key resources

Key reagents and resources used or developed in this study, including product codes, can be found in Supplementary Table 5.

### Quantification and statistical analysis

All statistical analysis was performed in Graphpad PRISM 9. IC_50_ values were calculated using the nonlinear regression function (variable slope—four parameters, least-squares regression). The statistical significance of IC_50_ changes was determined using ANOVA. All image analysis was performed in Zen 2 (Zeiss) and ImageJ/Fiji. Details of each analysis can be found in the corresponding figure legend.

### Reporting summary

Further information on research design is available in the Nature Portfolio Reporting Summary linked to this article.

## Supplementary information

Reporting Summary

Supplementary Table 1 Orthologues of known apicomplexan egress and invasion genes and putative egress and invasion genes of *B. divergens*.

Supplementary Table 2 Summary of selection drugs, resistance markers and transfection methods used for *B. divergens*.

Supplementary Table 3 A comparison of egress features across *B. divergens*, *P. falciparum* and *T. gondii*.

Supplementary Table 4 Primers used in this study.

Supplementary Table 5 Key resources.

Supplementary Video 1 8-Br-cGMP induced egress.

Supplementary Video 2 IC and EC without calcium plus saponin.

Supplementary Video 3 IC and EC with calcium plus saponin.

Supplementary Video 4 8-Br-cGMP induced egress of Cytochalasin D treated parasites.

Supplementary Video 5 ASP2 knockdown plus 8-Br-cGMP.

Supplementary Video 6 ASP3 knockdown plus 8-Br-cGMP.

## Source data

Source Data Fig. 3 Unprocessed western blots for Fig. 3.

Source Data Fig. 5 Statistical source data for Fig. 5.

Source Data Extended Data Fig. 3 Unprocessed gels for Extended Data Fig. 3d and unprocessed western blots for Extended Data Fig. 3f.

## Data Availability

The scRNA-seq (PRJNA803312) and bulk RNA-seq data (PRJNA804502) have been deposited at the National Center for Biotechnology Information (NCBI) Sequence Read Archive (SRA) and are publicly available. Source data are provided with this paper.

## Code availability

All code is available via GitHub at https://github.com/umbibio/Babesia_time_course and Zenodo at 10.5281/zenodo.17673515 (ref. ^88^).
