# Supplementary material for: Babesia divergens host cell egress is mediated by essential and druggable kinases and proteases
Source: Nat Microbiol. 2026 Jan 27;11(2):492–506. doi: 10.1038/s41564-025-02238-7 (PMC12872469; doi:10.1038/s41564-025-02238-7)
Supplement: Supplementary file 3 — Summary of selection drugs, resistance markers and transfection methods used for B. divergens. [file 41564_2025_2238_MOESM3_ESM.pdf]

Selection Markers				
**Resistance gene**	**Selection drug**	**IC_{50}**	**Concentration used for selection**	**Positive transfection**
hDHFR	Pyrimethamine	>500 nM	N/A	N/A
	WR99210	43 nM	100 nM	No
BSD	Blasticidin-S	12 µg/ml	20 µg/ml	**Yes**
PAC	Puromycin	0.22 µg/ml	0.5 µg/ml	**Yes (less reliable)**
yDHODH	DSM-1	>100 µM	N/A	N/A
	Atovaquone	24 nM	100 nM	No (spontaneous resistance)
HYG	Hygromycin B	>500 µg/ml	N/A	N/A

Transfection Methods				
**Transfection System**	**Protocol**	**DNA transfected**	**Parasites transfected**	**Time to reach 1% parasitemia**
Bio-rad gene pulser II	310 V, 950 µF, ∞ ohms. Cytomix	100 µg	200 µl packed iRBCs, ~20-30% parasitemia	21 days
Amaxa 4D nucleofection	FP158 / P3 solution 100 µl	10 µg	Free merozoites From 1.2 ml packed iRBCs, ~20-30% parasitemia	9 days
Amaxa 4D nucleofection	FP158 / P3 solution 100 µl	10 µg	20 µl packed iRBCs ~20-30% parasitemia	14 days
