# Supplementary material for: Babesia divergens host cell egress is mediated by essential and druggable kinases and proteases
Source: Nat Microbiol. 2026 Jan 27;11(2):492–506. doi: 10.1038/s41564-025-02238-7 (PMC12872469; doi:10.1038/s41564-025-02238-7)
Supplement: Supplementary file 4 — A comparison of egress features across B. divergens, P. falciparum and T. gondii. [file 41564_2025_2238_MOESM4_ESM.pdf]

	Babesia divergens (asexual)	*Plasmodium* spp. (asexual)	*Toxoplasma gondii* (tachyzoites)
**Host cell**	Enucleated RBC	Enucleated RBC	Nucleated cells
**Cell division**	Binary fission (2 daughter cells)	Schizogony (~8-32 daughter cells)	Endodyogeny (2 daughter cells)
**Apicoplast**	Present	Present	Present
**Secretory organelles**	Present	Present	Present
**Parasitophorous vacuole**	Degraded soon after invasion	Maintained throughout replication	Maintained throughout replication
**Egress throughout replication**	Can be induced to egress throughout replication but more strongly primed in mature parasites	Egress is limited to narrow window at the end of replication	Egress can be induced through majority of replication, with reduced efficiency during S and M/C phase
**Motility**	Required for escape from host cell	Not required for egress	Required for escape from host cell
**Response to extracellular environment**	Exposure to high calcium induces motility	Can sense serum albumin, potassium, calcium and lipids to induce egress/motility	Can sense lipids and potassium, however, role in egress and invasion remain unclear
**Egress coupling to division**	Egress can occur after 1 or more rounds of replication (typically 1-2 rounds). Inhibition of egress results in additional rounds of intracellular replication	Egress occurs at the end of the replication cycle (always a single round). Inhibition of egress stalls replication	Egress can occur after 1 or more rounds of replication (typically >4 rounds). Inhibition of egress results in additional rounds of intracellular replication
**Aspartyl proteases**	ASP2 is required for invasion and putatively process rhoptry proteins. ASP3 is required for egress and invasion, and putatively processes microneme proteins.	PMIX (Rhoptry) and PMX (microneme) are required for invasion, and egress and invasion, respectively.	Single ASP3 that processes rhoptry and microneme proteins in post-golgi compartment
**cGMP-dependent kinase (PKG)**	Required for egress and invasion	Required for egress and invasion	Required for egress and invasion
**Calcium-dependent kinases (CDPKs)**	CDPK4 is required for egress. It's role in invasion remains unclear.	Multiple CDPK's have overlapping roles in egress, motility and invasion. Functional orthology is not directly conserved between apicomplexan parasites.	Multiple CDPK's have overlapping roles in egress, motility and invasion. Functional orthology is not directly conserved between apicomplexan parasites.
**cAMP-dependent kinase (PKAc)**	2 PKAc genes. Unclear role. PKAc1 putatively	1 PKAc gene. PKAc1 is required for invasion but not egress.	3 PKAc proteins. PKAc1 represses premature egress after invasion.

	represses egress and is required for invasion.		
**Perforin-like proteins (PLPs)**	Unclear role. Lytic factors (putatively including PLP’s) are likely required for RBC lysis	Not required for egress (is required for gametocyte egress)	PLP1 is required for efficient egress
