# Supplementary material for: Babesia divergens host cell egress is mediated by essential and druggable kinases and proteases
Source: Nat Microbiol. 2026 Jan 27;11(2):492–506. doi: 10.1038/s41564-025-02238-7 (PMC12872469; doi:10.1038/s41564-025-02238-7)
Supplement: Supplementary file 5 — Primers used in this study. [file 41564_2025_2238_MOESM5_ESM.pdf]

Primer name	Primer sequence (5'-3')	Primer function
BE-8	ATGACTCGAGGCTAGCGTTTGGCACTGTTGCTCC	Fw Bd. EF1 5'UTR
BE-9	ATGAGGATCCGATTACAAGGTACCATTAATCTGCA AAGG	Rv Bd. EF1 5'UTR
BE-21	ATGAAAGCTTGTTATACCGCTTTAAGCTGG	Fw Bd DHFR 3'UTR
BE-22	ATGAGAATTCGTTGCGATATATATTGACTCTGTTTC	Rv Bd DHFR 3'UTR
BE-32	ATGAACTAGTATATGTAATATAAAGGCTATGTGTAT GCATC	Fw Bd Hsp90 3'UTR
BE-33	ATGAGCGGCCGCCGCTGATGTGGCACTAGC	Rv Bd Hsp90 3'UTR
BE_124	gcagattaatggtaccttgtaatcctcgagATGGACTATAAGGAC CACGACGG	Fw Cas9
BE_125	acacatagcctttatattacatatactagtTTACTTTTTCTTTTTTG CCTGGCCG	Rv Cas9
BE_154	ggttCTCCAGTCACAAGTTCTGTT	Fw Bd cas9 guide PKG gatekeeper mutation
BE_155	aaacAACAGAACTTGTGACTGGAG	Rv Bd cas9 guide PKG gatekeeper mutation
BE_213	tgggatatattagccgtatcGTTTGGCCGATGTATGGTCCTT TGG	Fw PKG HR1 for tagging
BE_214	acatcgtaagggtaaccggtAAAATCAAGTTCCCAgTCcTCG TCTGTGAGaTCTTC	Rv PKG HR1 for tagging
BE_215	GAAATCACATGATCTgtcgacACATAGTTATGAAGCA ACTGATTGTGAGC	Fw PKG HR2 for tagging
BE_216	ctcactatagaattcttaattaaCAAACGTGACAGATGGCATC TGAACC	Rv PKG HR2 for tagging
BE_221	ggttAGACCTCACAGACGAAGATT	Fw Cas9 Guide for PKG tagging
BE_222	aaacAATCTTCGTCTGTGAGGTCT	Rv Cas9 Guide for PKG tagging
BE_494	tgggatatattagccgtatcctaggCGGATAATGCTCCAGCAG TCG	Fw HR1 BdPLP1
BE_495	acatcgtaagggtaaccggtTTGTTTGCGGTCTGATAGTTC TGGT	Rv HR1 BdPLP1
BE_496	gaaatcacatgatctgtcgacTCGCACTGTCATGTACCATAG ATTTAAACG	Fw HR2 BdPLP1
BE_497	ctcactatagaattcttaattaaTGATGTGACTCTTGCACATAC AGC	Rv HR2 BdPLP1
BE_500	tgggatatattagccgtatcctaggcagAATGAGATAGCGGTAT CCTGC	Fw HR1 BdPLP3
BE_501	acatcgtaagggtaaccggtTGTTTTAATGTTGTTaGTgTCG GCAC	Rv HR1 BdPLP3
BE_502	gaaatcacatgatctgtcgacCATCCAAAAGTATCACAATCA CGACG	Fw HR2 BdPLP3
BE_503	ctcactatagaattcttaattaaACAGCAGAAGACTAAGTGTT CATACG	Rv HR2 BdPLP3
BE_506	tgggatatattagccgtatcctaggCCTTTGAAGTGCTAAACAT GGAGG	Fw HR1 BdCDPK5
BE_507	acatcgtaagggtaaccggtGTCTAATGTGGCTACATCTCC GG	Rv HR1 BdCDPK5
BE_508	gaaatcacatgatctgtcgacCTCATTCAAACTAAGATGTAC ACTACGTTTACAC	Fw HR2 BdCDPK5
BE_509	ctcactatagaattcttaattaaTGCATTGGGTTCCCTTTTCCC	Rv HR2 BdCDPK5
BE_512	tgggatatattagccgtatcctaggTCACGCAGAAACTCTCACA AGC	Fw HR1 BdCDPK4

BE_513	acatcgtaagggtaaccggtAACgAATCTTGTCAACATGGCC	Rv HR1 BdCDPK4
BE_514	gaaatcacatgatctgtcgacAAATTTTTCAATAAATGGCAATAGCTTATCAC	Fw HR2 BdCDPK4
BE_515	ctcactatagaattcttaattaaGTGTAGATTTTGTCTTTGCCTATCG	Rv HR2 BdCDPK4
BE_516	ggttCAAGGCCATGTTGACAAGAT	Guide fw BdCDPK4
BE_517	aaacATCTTGTCAACATGGCCTTG	Guide rv BdCDPK4
BE_518	tgggatatattagccgtatcctaggGCGACTATAAAGCTGACAGACTTTGG	Fw HR1 BdCDPK7
BE_519	acatcgtaagggtaaccggtCTCCTCAACaGTGCCCAG	Rv HR1 BdCDPK7
BE_520	gaaatcacatgatctgtcgacATGCCTAAGCGCCGTCG	Fw HR2 BdCDPK7
BE_521	ctcactatagaattcttaattaaGTGACAGAGATTCAAGCTTGTACAGG	Rv HR2 BdCDPK7
BE_524	tgggatatattagccgtatcctaggGCACATACACTCTATGTGGTACTCC	Fw HR1 BdPKAc1
BE_525	acatcgtaagggtaaccggtCCAGTTgTCaAAGGGGTCGG	Rv HR1 BdPKAc1
BE_526	gaaatcacatgatctgtcgacTACTGTGAAAGTTCACGCAGAATTCC	Fw HR2 BdPKAc1
BE_527	ctcactatagaattcttaattaaTGCATGAGTTGTTCAGAGTCAAACG	Rv HR2 BdPKAc1
BE_530	tgggatatattagccgtatcctaggAACGCACGATTACCTTGCTCC	Fw HR1 BdPKAc2
BE_531	acatcgtaagggtaaccggtAACGTAAACGGCTCTGAGAAACG	Rv HR1 BdPKAc2
BE_532	gaaatcacatgatctgtcgacCGGCATCATATTTTGCCTGAAATTTGG	Fw HR2 BdPKAc2
BE_533	ctcactatagaattcttaattaaCCTGCACGGGAAAATGTCGC	Rv HR2 BdPKAc2
BE_534	ggttTCAGAGCCGTTTACGTTTAG	Guide fw BdPKAc2
BE_535	aaacCTAAACGTAAACGGCTCTGA	Guide rv BdPKAc2
BE_536	ACCGGTTACCCTTACGATGTTCCTGACTATGC	Fw 1xHA (Bd) for Knockdown tags. Universal for all.
BE_537	gtcgacAGATCATGTGATTTCTCTTTGTTCAAGG	Rv glmS (Bd) for Knockdown tags. Universal for all.
BE_550	CCTAGGATACGGCTAATATATCCCATC	Rv Bd U6 terminator – for universal use with guide PCR
BE_551	CAAGTATCGATACAGGGAGGTTGACAGTGCGATCATTGTTTGgttttagagctaGAAAtagcaagttaaaataagg	PLP1 guide
BE_552	CAAGTATCGATACAGGGAGGTTTGTTTTAATGTTGTTCGTATgttttagagctaGAAAtagcaagttaaaataagg	PLP3 guide
BE_553	CAAGTATCGATACAGGGAGGTTCAAGGCCATGTTGACAAGATgttttagagctaGAAAtagcaagttaaaataagg	CDPK4 guide
BE_554	CAAGTATCGATACAGGGAGGTTTTGAATGAGTTAGTCTAATGgttttagagctaGAAAtagcaagttaaaataagg	CDPK5 guide
BE_555	CAAGTATCGATACAGGGAGGTTGCTTAGGCATCACTCCTCAAgttttagagctaGAAAtagcaagttaaaataagg	CDPK7 guide
BE_556	CAAGTATCGATACAGGGAGGTTACAGTATCACCAGTTATCGAgttttagagctaGAAAtagcaagttaaaataagg	PKAc1 guide
BE_557	CAAGTATCGATACAGGGAGGTTTCAGAGCCGTTTACGTTTAGgttttagagctaGAAAtagcaagttaaaataagg	PKAc2 guide PCR (or Gibson ssODN)
BE_558	CAAGTATCGATACAGGGAGGTTATCTGCTGAACAAGGTTACAgttttagagctaGAAAtagcaagttaaaataagg	PhoD guide PCR (or Gibson ssODN)

BE_563	CAAGTATCGATACAGGGAGGTTGAGTCTATTTTGC ATTATTCgttttagagctaGAAAtagcaagttaaaataagg	Guide Asp2
BE_564	tgggatatattagccgtatcctaggTTCGGATGATGCCAGTTGT CG	Fw HR1 Asp2
BE_565	acatcgtaagggtaaccggtTTTTGCATTATTCCGGTGCTT GG	Rv HR1 Asp2
BE_566	gaaatcacatgatctgtcgacACTCTTAATTTCAACATGTCG TGCG	Fw HR2 Asp2
BE_567	ctcactatagaattcttaattaaGGCAACCACTTCAAAGTTTGC	Rv HR2 Asp2
BE_568	CAAGTATCGATACAGGGAGGTTTATGCATCCATAG GATCATAgttttagagctaGAAAtagcaagttaaaataagg	Guide Asp3
BE_569	tgggatatattagccgtatcctaggAGTTGTTCGCGAGCACTAT TGG	Fw HR1 Asp3
BE_570	acatcgtaagggtaaccggtGTAGTGTCTTGACGATGCAAT CC	Rv HR1 Asp3
BE_571	gaaatcacatgatctgtcgacTGTATATATACtATATGATCCT ATGGATGCATAC	Fw HR2 Asp3
BE_572	ctcactatagaattcttaattaaACAGCTACATGAGTCAGTAG AAAGG	Rv HR2 Asp3
BE_573	CAAGTATCGATACAGGGAGGTTTAATTAGTTATGTT TTAATAgttttagagctaGAAAtagcaagttaaaataagg	Guide DPAP1
BE_574	tgggatatattagccgtatcctaggCATACCTAACAGGAAAAC GCATGC	Fw HR1 DPAP1
BE_575	acatcgtaagggtaaccggtTGTTTTAATATGGTTGTGTACA TCGTGTAG	Rv HR1 DPAP1
BE_576	gaaatcacatgatctgtcgacCTAATTAATCGTGTTTTAAAAT GCAACACGAC	Fw HR2 DPAP1
BE_577	ctcactatagaattcttaattaaGTAATGCTCCTTTATCTCCTT CCACATG	Rv HR2 DPAP1
BE_596	ACCTGAAACTCACGGACTTCG	Fw test PKAc1 tag Integration
BE_597	CGAACCACTCCCGTATATGTCC	Rv test PKAc1 tag Integration
BE_598	ACTTTGGTTTTGCCAAGCACG	Fw test PKAc2 tag Integration
BE_599	GATGGCTGGTCATACAGTGC	Rv test PKAc2 tag Integration
BE_600	GGAAAGGAAAAGTGTGTGGCG	Fw test PLP1 tag Integration
BE_601	TCGCCTCAAACGCGGATTCC	Rv test PLP1 tag Integration
BE_602	AAGACAAACATgtaaggagttgtgc	Fw test PLP3 tag Integration
BE_603	TCTCGTATGCCAGTATGAAGTGC	Rv test PLP3 tag Integration
BE_604	CATTATGTTTGGAGGTGCTGATGC	Fw test Asp2 tag Integration
BE_605	CACAACAAGTCTCAAGATGAATAGGAGC	Rv test Asp2 tag Integration
BE_606	GTTTGGTGGAGTGGATCCAAGG	Fw test Asp3 tag Integration
BE_607	AGGTTTCATGAAGCTTTTGGTTTCG	Rv test Asp3 tag Integration
BE_608	ACCTGCGATATGTTCAACCAGG	Fw test DPAP1 tag Integration

BE_609	GATTTACTTCTTCTGCACGAGTGC	Rv test DPAP1 tag Integration
BE_610	CATCAAACAGGTGCTCAGTGG	Fw test CDPK4 tag Integration
BE_611	CAAAAGGCTGCATCTGGTATGC	Rv test CDPK4 tag Integration
BE_612	GTAATGCGGCAGATCTTTTCTGC	Fw test CDPK5 tag Integration
BE_613	GAACCACTTCCAAACCCCTGG	Rv test CDPK5 tag Integration
BE_614	TCGTGAATTTTACGTGGCTTCTAGG	Fw test CDPK7 tag Integration
BE_615	ATCAGTTGGTCAACACGTGGC	Rv test CDPK7 tag Integration
BE_179	GCACGGTTGGTTTTTTAATACACCG	Fw test PKG gatekeeper Integration
BE_180	ACCAAGTATGATTTCAGGTGCC	Rv test PKG gatekeeper Integration
Synthesis 1	gagtcaatatatatcgcaacgaattctctagaGTGGTGTTAATTCA TCGCTAGTAACCATATACGAGGGCATAGACAAGTC CTTCCACACGTGCATAGCCGCATTGGCTAGTAAGC TAAATATAGAGCAGATTTATGTCACCTTCAATTATT CTTTCTCAGCATGAATGTCACCTGTTTATGTCATAG TACGGCATAAACAGGCACGGCAATGGCACGGTCA CCTTCTACATCACCAAACTACAATTGAAGTACAACA ACAAAGTCAACTAATGAAGGTCTCCTCTATATCAAA GAGCGAGTCGTGTAGAGATAGTGATCCTCCGAAAC TATTAAATGAAACCAGATATAGTGAAATGCTGTCCA ACGTGCAGAAATAACCTTGCTTCATGATGTTTGGC AGCTCCCATATAGATCTGTACCATTTCAAACATTTT TATATGGCCATTTTTCTATTATATGTCGCATTGATC CTTATCCTTAATTTTGCATCCCCACGGTGGTACCTG TTGAGTGAGAGTCCCACTCCCCACAAGTATCGATA CAGGGAGGTTggGTCTTCgaGAAGACctgttttagagctaG AAAtagcaagttaaaataaggctagtccgttatcaacttgaaaaagtggc accgagtcggtgcTTTTTTTTTTGCATCTTTGCACAGAAT ATATCAATGATGGGATATATTAGCCGTATCCTAGGT ATAGGAACTTTCGGGACCGTTAAATTGGTAGAGCA TGAGGCCACAGGTGTAAGATTTGCTTTGAAGTGTG TTAGTAGGAAATGTATCCGTGCACTCAAGCAAGAG AAGCATATCAAGTTGGAAAGAGAAATAATGGCTCA GAATGACCATCCATTCATCGTTCAACTAGGTAATTG GTGCCGTGCCTTAAGCTAAATACTCTGCAGTAAAA ACATTcAAaGAcGCTGATAATGTCTACTTtCTAcaAGA ACTaGTGACTGGAGGTGAATTGTACGATGCAATCA GAAAAATTGGGTTGCTTTCGAGGTCTCAAGCACAA TTTTACATAGCATCTATCGTCCTAGCATTCGAGTAT TTGCATGAAAGACAAATCGCATATCGAGTAGGTTC AGAAGCTAAATGATTAATGCTTTCATAGGATTTGAA ACCTGAAAATATACTTCTCGATGAACAGGGCTATAT CAAACTGATTGATTTCGGATGTGCAAAAAAAATTAA AGAGGGCCTTAATTAAgaattctatag	BdU6 promoter-guide cloning -PKG gatekeeper HR
