# Supplementary material for: Babesia divergens host cell egress is mediated by essential and druggable kinases and proteases
Source: Nat Microbiol. 2026 Jan 27;11(2):492–506. doi: 10.1038/s41564-025-02238-7 (PMC12872469; doi:10.1038/s41564-025-02238-7)
Supplement: Supplementary file 6 — Key resources. [file 41564_2025_2238_MOESM6_ESM.pdf]

Table S5 – Key resources

REAGENT or RESOURCE	SOURCE	IDENTIFIER
**Antibodies**		
Anti-Histone H3 antibody	Abcam	ab1791; RRID:AB_302613
Anti-HA 3F10	Sigma (Roche)	12158167001; RRID:AB_390918
**Bacterial and virus strains**		
E. coli XL10-Gold Ultracompetent cells (all cloning)	Agilent	200315
**Chemicals, peptides, and recombinant proteins**		
**8-Bromoguanosine 3′,5′-cyclic monophosphate sodium salt (8-Br-cGMP)**	Sigma-Aldrich	B1381
SYBR™ Green I Nucleic Acid Gel Stain	Invitrogen	S7563
KT5720	Sigma-Aldrich	K3761
Heparin sodium salt from porcine intestinal mucosa	Sigma-Aldrich	H3393
**8-Bromoadenosine 3′,5′-cyclic monophosphate sodium salt (8-Br-cAMP**)	Sigma-Aldrich	B7880
1-[6-[((17β)-3-Methoxyestra-1,3,5[10]-trien-17-yl)amino]hexyl]-1H-pyrrole-2,5-dione (U-73122)	Sigma-Aldrich	U6756
1,2-Bis(2-aminophenoxy)ethane-N,N,N′,N′-tetraacetic acid tetrakis(acetoxymethyl ester) **(**BAPTA-AM)	Sigma-Aldrich	A1076
**Cytochalasin D**	Sigma-Aldrich	C8273
E64	Sigma-Aldrich	E3132
Compound-1 (4-[2-(4-fluorophenyl)-5-(1-methylpiperidine-4-yl)-1H pyrrol-3-yl]-pyridine)	A gift from Dr. Jeffrey Dvorin (Boston Children's Hospital)	
R59022 (Diacylglycerol Kinase Inhibitor I)	Sigma-Aldrich	D5919
Phenylmethylsulfonyl fluoride (PMSF)	Sigma-Aldrich	78830
***N*$_{\alpha}$-Tosyl-L-lysine chloromethyl ketone hydrochloride (TLCK)**	Sigma-Aldrich	T7254
N-p-Tosyl-L-phenylalanine chloromethyl ketone (TPCK)	Sigma-Aldrich	P5318
Pepstatin A	Sigma-Aldrich	P5318
Chymostatin	Sigma-Aldrich	C7268

E64d	Selleckchem	S7393
ALLN (Calpain inhibitor 1)	Sigma-Aldrich (Calbiochem)	208719
H89	Sigma-Aldrich	B1427
BIPPO 5-benzyl-3-isopropyl-1H-pyrazolo[4,3-d]pyrimidin-7(6H)-one	A gift from Dr. Jeffrey Dvorin (Boston Children's Hospital).	
A23187	Sigma-Aldrich	C7522
Propranolol hydrochloride	Sigma-Aldrich	P0884
ML10	A gift from Dr. Simon Osborne (LifeArc)	
Saponin	Calbiochem	558255
Alexa Fluor™ 488 Phalloidin	Invitrogen	A12379
Shld1	Synthesized as per [88]	
TRIzol®	Invitrogen	15596026
D-(+)-Glucosamine hydrochloride (GlcN)	Sigma-Aldrich	G1514
Blasticidin-S	Invivogen	ant-bl-10p
WM382	GLPBIO	GC70146
UCB7362 hydrochloride	TargetMOL	T69567L
**Critical commercial assays**		
DNeasy Blood and Tissue Kit	Qiagen	69504
RNeasy Mini spin-columns	Qiagen	74106
**P3 Primary Cell 4D-Nucleofector™ Kit**	Lonza	V4XP-3024
**Deposited data**		
Bulk synchronous RNAseq	NCBI SRA	PRJNA804502
Single-cell RNAseq	NCBI SRA	PRJNA803312
Code	GitHub and Zenodo	https://github.com/umbibio/Babesia_time_course<br><br>DOI: https://doi.org/10.5281/zenodo.17673515
**Experimental models: Cell lines**		

Babesia divergens strain Rouen 1987	Kindly provided by Kirk Deitsch and Laura Kirkman (Weill Cornell Medical College)	
Babesia bovis strain MO7	Kindly provided by David Allred (University of Florida)	
Babesia bigemina strain	Kindly provided by David Allred (University of Florida)	
**Oligonucleotides**		
See supplementary table 4 for primers and synthesis products		
**Recombinant DNA**		
pBdEF1-GFP-BSD	This study	
pBdEF1-Cas9-BSD-PKG-T651Q	This study	
pBdEF1-Cas9-BSD-CDPK4-HA-DD-glmS	This study	
pBdEF1-Cas9-BSD-CDPK5-HA-DD-glmS	This study	
pBdEF1-Cas9-BSD-CDPK7-HA-DD-glmS	This study	
pBdEF1-Cas9-BSD-PKAc1-HA-DD-glmS	This study	
pBdEF1-Cas9-BSD-PKAc2-HA-DD-glmS	This study	
pBdEF1-Cas9-BSD-ASP2-HA-DD-glmS	This study	
pBdEF1-Cas9-BSD-ASP3-HA-DD-glmS	This study	
pBdEF1-Cas9-BSD-DPAP1-HA-DD-glmS	This study	
pBdEF1-Cas9-BSD-PLP1-HA-DD-glmS	This study	
pBdEF1-Cas9-BSD-PLP3-HA-DD-glmS	This study	
**Software and algorithms**		
Prism version 9 and 10	GraphPad	
ImageJ/Fiji version 2.0	https://imagej.net/software/fiji/	
Zen 2	Zeiss	
VEuPathDB release 46 (PiroplasmaDB/PlasmoDB/ToxoDB)	https://veupathdb.org/veupathdb/app	
FlowJo version 10	FlowJo	
**Other**		
8/18-well glass bottom imaging chamber	Ibidi	80827/81817

**1.2 µM Pall Acrodisc® Sterile Syringe Filters with Supor® Membrane**	Pall	4656
