# Supplementary figures and images for: Babesia divergens host cell egress is mediated by essential and druggable kinases and proteases
Source: Nat Microbiol. 2026 Jan 27;11(2):492–506. doi: 10.1038/s41564-025-02238-7 (PMC12872469; doi:10.1038/s41564-025-02238-7)

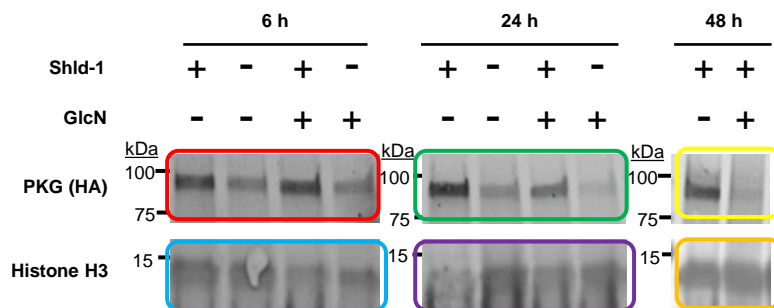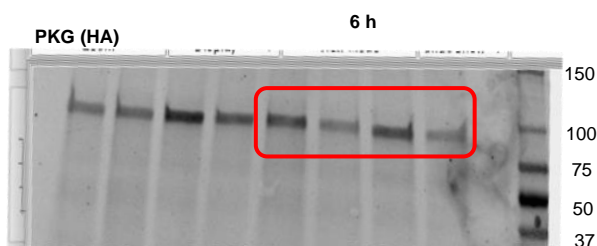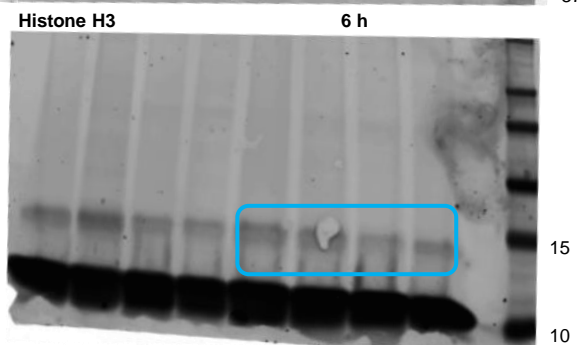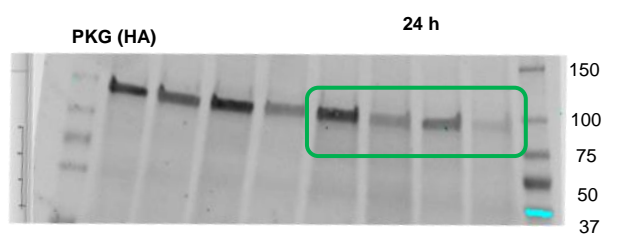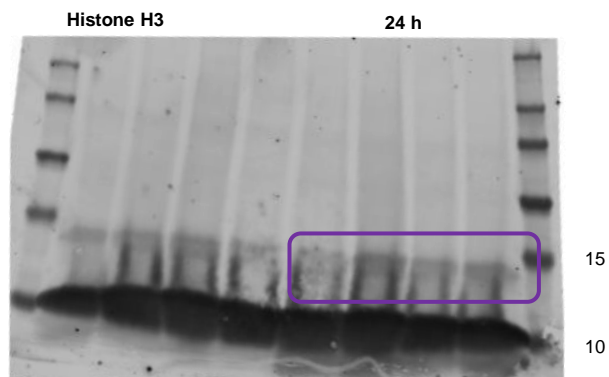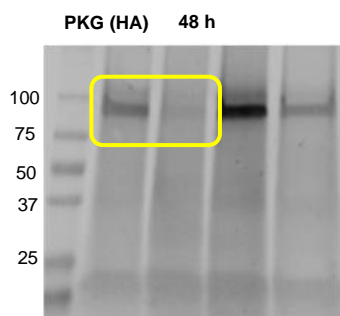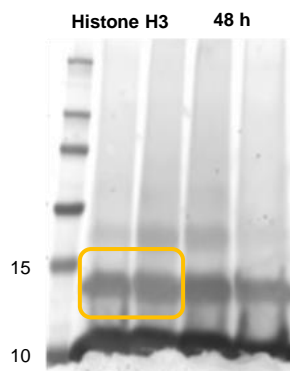

Supplement: Supplementary file 13 — Unprocessed western blots for Fig. 3. [file 41564_2025_2238_MOESM13_ESM.pdf]

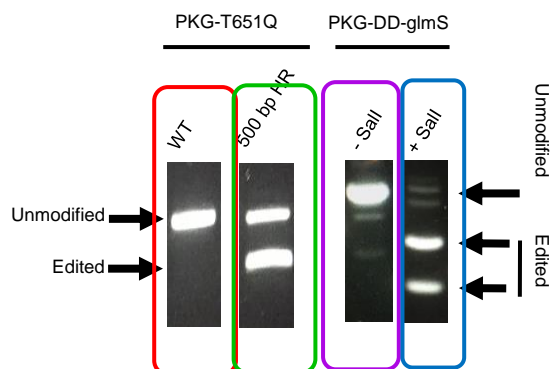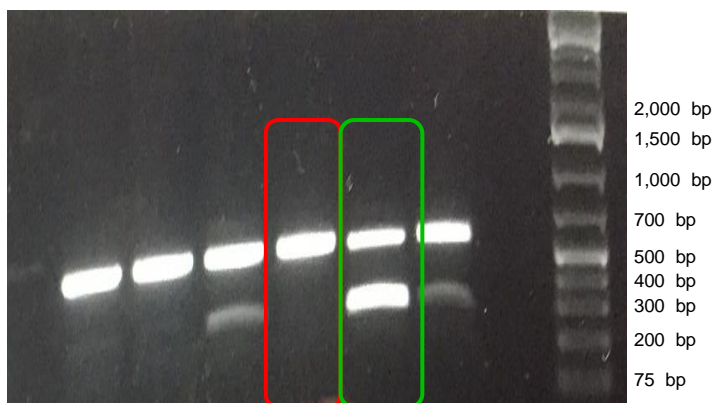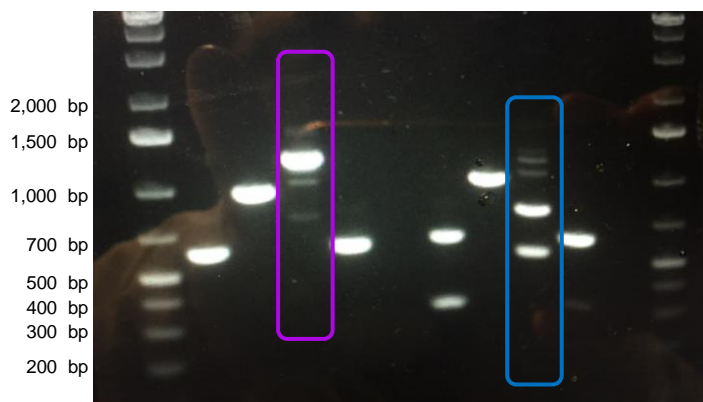

Supplement: Supplementary file 15 — Unprocessed gels for Extended Data Fig. 3d and unprocessed western blots for Extended Data Fig. 3f. [file 41564_2025_2238_MOESM15_ESM.zip › ED3 Source Data/23020296A-Z_Source_data_for_extended_data_figure_3D_.pdf]

|        | ASP2 (6 h) |   | ASP3 (6 h) |   | DPAP1 (48 h) |   |
|--------|------------|---|------------|---|--------------|---|
| Shld-1 | +          | - | +          | - | +            | - |
| GlcN   | -          | + | -          | + | -            | + |

CDPK4 (48 h)

NOT PUBLISHED

|   |   |
|---|---|
| + | - |
| - | + |

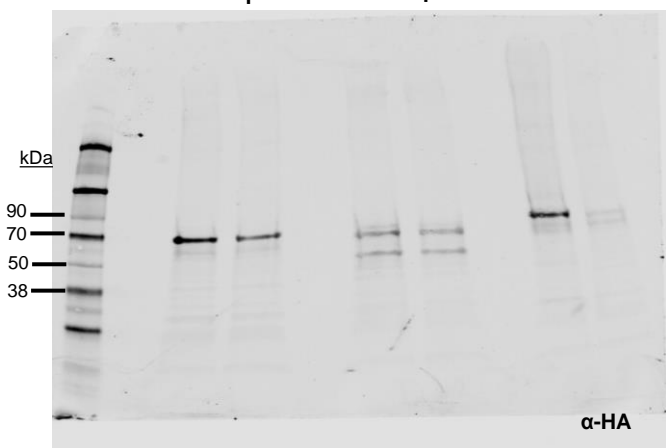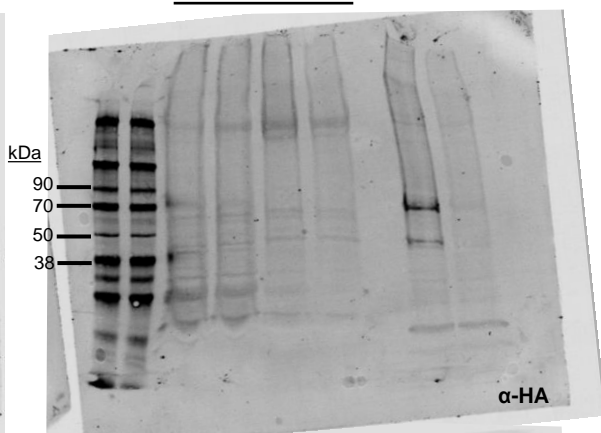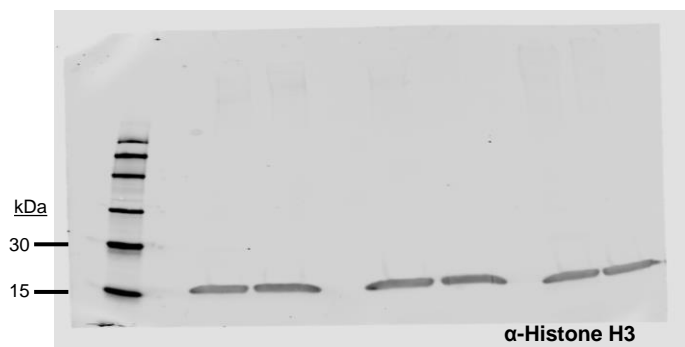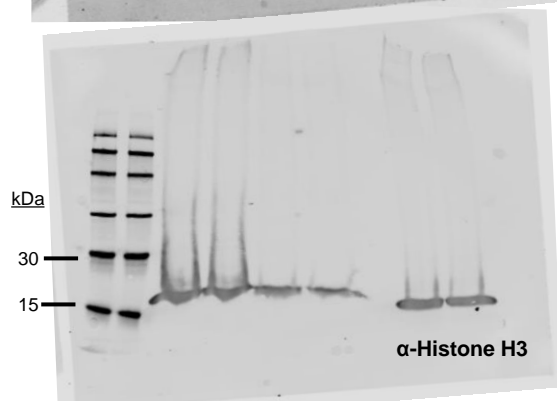

Supplement: Supplementary file 15 — Unprocessed gels for Extended Data Fig. 3d and unprocessed western blots for Extended Data Fig. 3f. [file 41564_2025_2238_MOESM15_ESM.zip › ED3 Source Data/23020296A-Z_Source_data_for_extended_data_figure_3F_.pdf]
